# Estrogen-related receptor alpha directly binds to p53 and cooperatively controls colon cancer growth through the regulation of mitochondrial biogenesis and function

**DOI:** 10.1186/s40170-020-00234-5

**Published:** 2020-12-10

**Authors:** Humberto De Vitto, Joohyun Ryu, Ali Calderon-Aparicio, Josh Monts, Raja Dey, Abhijit Chakraborty, Mee-Hyun Lee, Ann M. Bode, Zigang Dong

**Affiliations:** 1 The Hormel Institute, University of Minnesota, 801 16th Avenue NE, Austin, 55912 USA; 2 Department of Pathophysiology, Zhengzhou University School of Medicine, 40 North Road, 27 District University, Zhengzhou, 450052 China

**Keywords:** ERRα, p53-deficient, Mitochondrial biogenesis, Mitochondrial oxidative phosphorylation (mtOxPhos), PDX colon cancer model, Apoptosis

## Abstract

**Background:**

Of the genes that control mitochondrial biogenesis and function, *ERRα* emerges as a druggable metabolic target to be exploited for cancer therapy. Of the genes mutated in cancer, *TP53* remains the most elusive to target. A clear understanding of how mitochondrial druggable targets can be accessed to exploit the underlying mechanism(s) explaining how p53-deficient tumors promote cell survival remains elusive.

**Methods:**

We performed protein-protein interaction studies to demonstrate that ERRα binds to p53. Moreover, we used gene silencing and pharmacological approaches in tandem with quantitative proteomics analysis by SWATH-MS to investigate the role of the ERRα/p53 complex in mitochondrial biogenesis and function in colon cancer. Finally, we designed in vitro and in vivo studies to investigate the possibility of targeting colon cancers that exhibit defects in p53.

**Results:**

Here, we are the first to identify a direct protein-protein interaction between the ligand-binding domain (LBD) of ERRα and the C-terminal domain (CTD) of p53. ERRα binds to p53 regardless of p53 mutational status. Furthermore, we show that the ERRα and p53 complex cooperatively control mitochondrial biogenesis and function. Targeting ERRα creates mitochondrial metabolic stresses, such as production of reactive oxygen species (ROS) and mitochondrial membrane permeabilization (MMP), leading to a greater cytotoxic effect that is dependent on the presence of p53. Pharmacological inhibition of ERRα impairs the growth of p53-deficient cells and of p53 mutant patient-derived colon xenografts (PDX).

**Conclusions:**

Therefore, our data suggest that by using the status of the p53 protein as a selection criterion, the ERRα/p53 transcriptional axis can be exploited as a metabolic vulnerability.

**Supplementary Information:**

The online version contains supplementary material available at 10.1186/s40170-020-00234-5.

## Background

Reprogramming of energy metabolism is included in the select list of biological hallmarks acquired during the development of cancer [1, 2]. The adaptive metabolic responses make achieving clinical benefits difficult when targeting a single metabolic pathway [3–5]. Conversely, therapeutically exploiting these metabolic liabilities can render cancer cells more susceptible to treatment with potential and selective metabolic inhibitors [6–8]. For instance, several researchers have suggested that targeting mitochondrial bioenergetics and function to overcome drug resistance in cancer treatment could be therapeutically exploited, whereas the resistance mechanism in cancer might be associated with a shift toward an increased mtOxPhos status [9–14]. Mitochondrial biogenesis is a key component of adaptive activities needed for cancer cells to survive periods of metabolic stress [15, 16]. Therefore, mitochondrial turnover and mtOxPhos activity might be considered as metabolic requirements of tumor cells, and therefore, understanding the major regulators of mitochondrial biogenesis and function might guide the treatments that could be exploited for effective cancer therapy.

Given the importance of nuclear receptors (NRs) in steroid, retinoid, and thyroid hormone signaling networks, hypothesizing that NRs are implicated in a wide range of pathologies is tempting [17]. Historically, NRs serve as important targets for hormone therapies [18]. The estrogen-related receptor family (ERRα, ERRβ, and ERRγ) was first described as an orphan nuclear receptor family and was originally identified based on its homology to estrogen receptor alpha (ERα) [19, 20]. Based on Warburg’s prediction, cancer cells increase metabolic requirements to sustain their proliferation [21]. ERRα is emerging as a major transcription factor that regulates an extremely broad repertoire of mitochondrial and metabolic genes [12, 16, 22–27], making this druggable protein very attractive for cancer treatment [28].

The function of the ERRα and peroxisome proliferator-activated receptor gamma coactivator 1-alpha (PGC-1α) transcriptional axis in the metabolic reprogramming of cancer cells toward mitochondrial metabolism in breast, melanoma, ovarian, prostate, and colorectal tumors is well-known [12, 28–32]. However, whether different transcriptional networks exist, whereby ERRα could interact with important transcription factors (TFs) leading to the regulation of cancer metabolism and survival, is not clear.

*TP53* is known to be the most frequently altered gene in human cancer [33, 34]. Unlike other tumor suppressors, the vast majority of cancer-associated mutations in *TP53* are missense mutations or gain-of-function (GOF) mutations leading to single base-pair substitutions (SNP) in the translation of a different amino acid in that position of the full-length protein. These mutations provide several advantages for the mutant forms of p53, such as a prolonged half-life and dominant negative effects of the mutant forms of p53 on the remaining wild-type allele [35, 36]. Although, the p53 protein is one of the most studied targets in cancer biology, the science surrounding the role of p53 continues to intrigue scientists, especially the complexity of p53 protein status, functions, and networks [35, 37].

The role of the p53 protein in inhibiting cell growth by inducing cell cycle arrest and apoptosis is very well established [38, 39]. Recently, the p53 protein has been shown to influence multiple processes in metabolism, especially in response to metabolic stress [40–43]. Interestingly, targeting the mitochondrial uncoupling mechanism using niclosamide impaired the growth of p53-deficient cells and p53 mutant patient-derived ovarian xenografts [44]. Also, the status of the p53 protein (with GOF activity) has been shown to determine the p53’s ability to bind and regulate different protein networks, which control tumor metabolism and metastasis [45]. Indeed, the p53 mutant protein can drive the expression of key regulators of proliferation, invasion, and metastasis through its binding with several transcription factors, such as Yes-associated protein (YAP), PGC-1α, and nuclear factor erythroid 2–related factor 2 (NRF2) [46]. However, despite the advances in the discovery of new functions of p53 that are dependent on the status of the protein [47, 48], the metabolic network of p53 interactions with key regulators of mitochondrial metabolism that lead to colon cancer cell survival has not yet been determined.

In this study, for the first time, we provide indirect and direct evidence for the protein-protein interaction of the ERRα LBD and the p53 CTD, which occurs independently of p53 status. Mechanistically, we describe in depth how the ERRα and p53 complex cooperatively regulates mitochondrial biogenesis and mtOxPhos leading to colon cancer cell survival. Notably, our study reveals an unexpected metabolic vulnerability for targeting ERRα in p53-deficient (nonsense) cells compared to wild-type p53 colon cancer cells. Because ERRα belongs to a class of druggable NRs, our in vitro and in vivo studies suggest that the ERRα antagonist XCT790 might be considered as a potential drug to treat colon cancer patients with defective p53 status.

## Results

### ERRα directly binds to p53

**Fig. 1:**
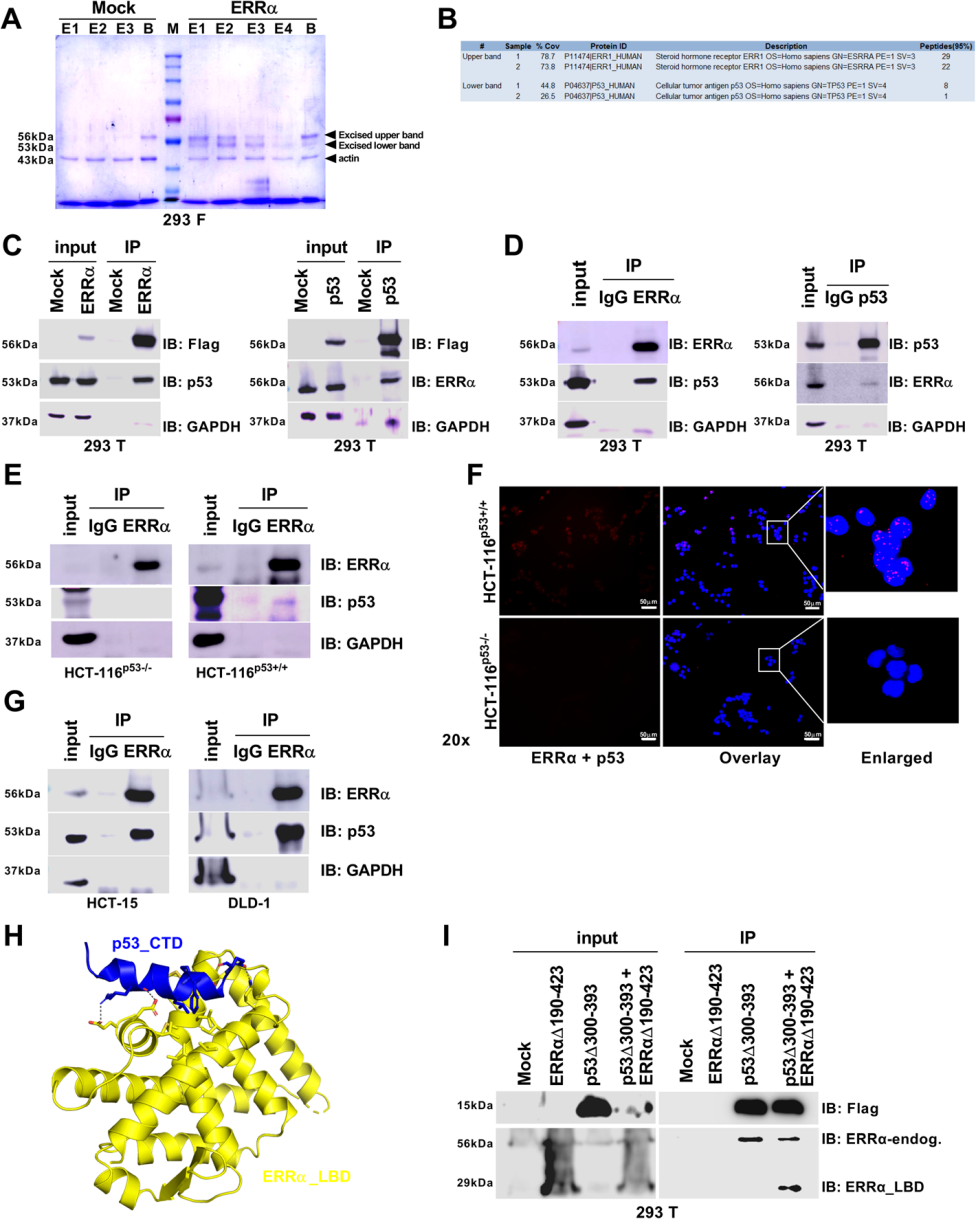
ERRα directly interacts with p53. **a** FreeStyle^TM^ 293-F cells transiently transfected with the pcDNA3 empty vector (mock) or pCMV flag ERRα were subjected to an affinity pull-down assay by using an ANTI-FLAG®M2 Affinity Gel. Proteins were stained with crystal violet (E1-E4, eluted fractions; B, beads). **b** After excision of the upper and lower bands from the gel, both samples were purified and further examined by LC-MS/MS. Overall, two independent experiments were performed in triplicate to identify ERRα (upper band) and p53 (lower band). **c** 293T cells were transiently transfected with the pcDNA3 empty vector (mock), pCMV flag ERRα, and pcDNA3 flag p53. Cells were then subjected to an affinity pull-down assay as for **a** and immunoblot (IB) analysis with monoclonal Anti-Flag®M2, anti-ERRα (*right*), anti-p53 (*left*), and anti-GAPDH as loading control for both. **d** Endogenous immunoprecipitation (IP) of ERRα (*left*) and p53 (*right*) was performed by using 293T cells followed by IB analysis with anti-ERRα (*right*), anti-p53 (*left*), and anti-GAPDH. **e** HCT-116^p53+/+^ (*right*) and HCT-116^p53−/−^ (*left*) cells were used to perform endogenous IP followed by IB analysis with anti-ERRα, anti-p53 (two different antibodies), and anti-GAPDH. **f** HCT-116^p53+/+^ (*top*) and HCT-116^p53−/−^ (*bottom*) cells were used to perform a proximity ligation assay followed by analysis by fluorescence microscopy. Scale bar, 50 μm. **g** HCT-15 (*left*) and DLD-1 (*right*) colon cancer cell lines were used to perform endogenous IP followed by IB analysis with anti-ERRα, anti-p53, and anti-GAPDH. **h** Modeling of the ERRα LBD interacting with the p53 CTD (ERRα LBD, yellow; p53 CTD, blue). See additional file 2: Figure S2 for more details. **i** 293T cells were transiently transfected with the pcDNA3 empty vector (mock), the pcDNA 3.1 ERRα LBDΔ190-423 with V5 Epitope/His tag, and the pcDNA3 p53 CTDΔ300-393 with a Flag tag. Cells were then subjected to an affinity pull-down assay as for **a**. This was followed by IB analysis with monoclonal Anti-Flag®M2 and the rabbit monoclonal anti-ERRα, which recognizes the endogenous level of the protein in the residues near the carboxy-terminus of the human ERRα protein. Data represent protein-protein interaction and were not quantified

The ERRα/PGC1α complex drives cancer cell survival through mitochondrial metabolic programs favoring tumor development [28, 49, 50]. Moreover, the p53/PGC1α complex drives tumor metabolism and metastasis through mitochondrial energy programs enabling mitochondria to cope with metabolic stress [45, 51]. However, the ability of both ERRα and p53 to influence cell fate decisions, such as survival and death, has not been fully investigated. To determine whether ERRα might interact with p53, we first used 293F cells to perform a flag-ERRα protein purification (Fig. 1a) followed by both liquid chromatography-tandem mass spectrometry (LC-MS/MS; Fig. 1b) and size exclusion chromatography (SEC; additional file Fig. S1A) to determine the potential protein-protein interaction between ERRα and p53 and the stability of this complex, respectively. With a considerable percentage of confidence, we found that ERRα interacts with p53 to form a stable protein complex (Fig. 1a, b and additional file Fig. S1B, C). This finding prompted us to determine whether this interaction occurs only in an artificial system through mammalian cell transfection or as an endogenous protein-protein interaction. First, using 293T cells followed by overexpression of the flag-ERRα protein and immunoprecipitation (IP)/immunoblot (IB) analysis, we confirmed the protein-protein interaction between ERRα and p53 in a transfection system (Fig. 1c). Importantly, we then performed an endogenous co-IP of ERRα and p53 using 293T cells and showed that ERRα indeed interacts with p53 in cells (Fig. 1d).

ERRα regulates an extremely broad repertoire of mitochondrial and metabolic genes, making it a very attractive target for cancer treatment [17]. To determine whether its binding to p53 could occur in cancer cells, we used isogenic human colon cancer cell lines HCT-116^p53+/+^ (wild-type p53) and HCT-116^p53−/−^ (p53-null background). We performed an endogenous co-IP/IB analysis and observed that the wild-type p53 protein interacted with ERRα, whereas the p53-deficient cells showed no interaction with ERRα (Fig. 1e). Next, we performed a proximity ligation assay (PLA) to confirm that ERRα binds to p53. In the presence of wild-type p53, a clear immunofluorescence (IF) staining was observed, whereas in p53-deficient cells the IF staining was completely absent (Fig. 1f). To expand our findings, we used p53 mutant (missense or GOF) colon cancer cell lines, DLD-1 and HCT-15, to perform an endogenous co-IP/IB analysis. Interestingly, we also showed a marked increased binding of ERRα to the mutant form of the p53 protein (Fig. 1g).

To further determine whether ERRα directly interacts with p53, we used computational techniques to identify the binding interface and build a model of the proposed physical interaction of both proteins. Results indicate that the ERRα LBD interacts with three potentially different sites of the p53 CTD (Fig. 1h and additional file Fig. S2A-H). To directly confirm the in silico prediction, we constructed two different truncated proteins, an ERRα LBDΔ190-423 with a V5 Epitope/His tag and a p53 CTDΔ300-393 with a Flag tag. Next, we performed a Flag purification of p53 CTDΔ300-393 followed by an affinity pull-down assay and IB analysis to show that p53 CTDΔ300-393 physically interacts with ERRα LBDΔ190-423 (Fig. 1i). These results provided unequivocal evidence showing that the LBD of ERRα directly binds to the CTD of p53. Notably, we have performed endogenous IP against ERRα in 293T cells (p53 wild-type) and a colon cancer cell line DLD-1 (p53 mutant), and we showed that PGC-1α does not act as a surrogate protein ligand of the ERRα/p53 complex (additional file Fig. S1D). Overall, the interaction of the two proteins seems to occur in a colon cancer scenario regardless of p53 protein status, which could be exploited as a therapeutic opportunity for targeting ERRα in different cancer-related mutant forms of p53.

### Gene silencing of *ERRα* decreases p53 expression impairing mitochondrial biogenesis and mtOxPhos in colon cancer cells

**Fig. 2:**
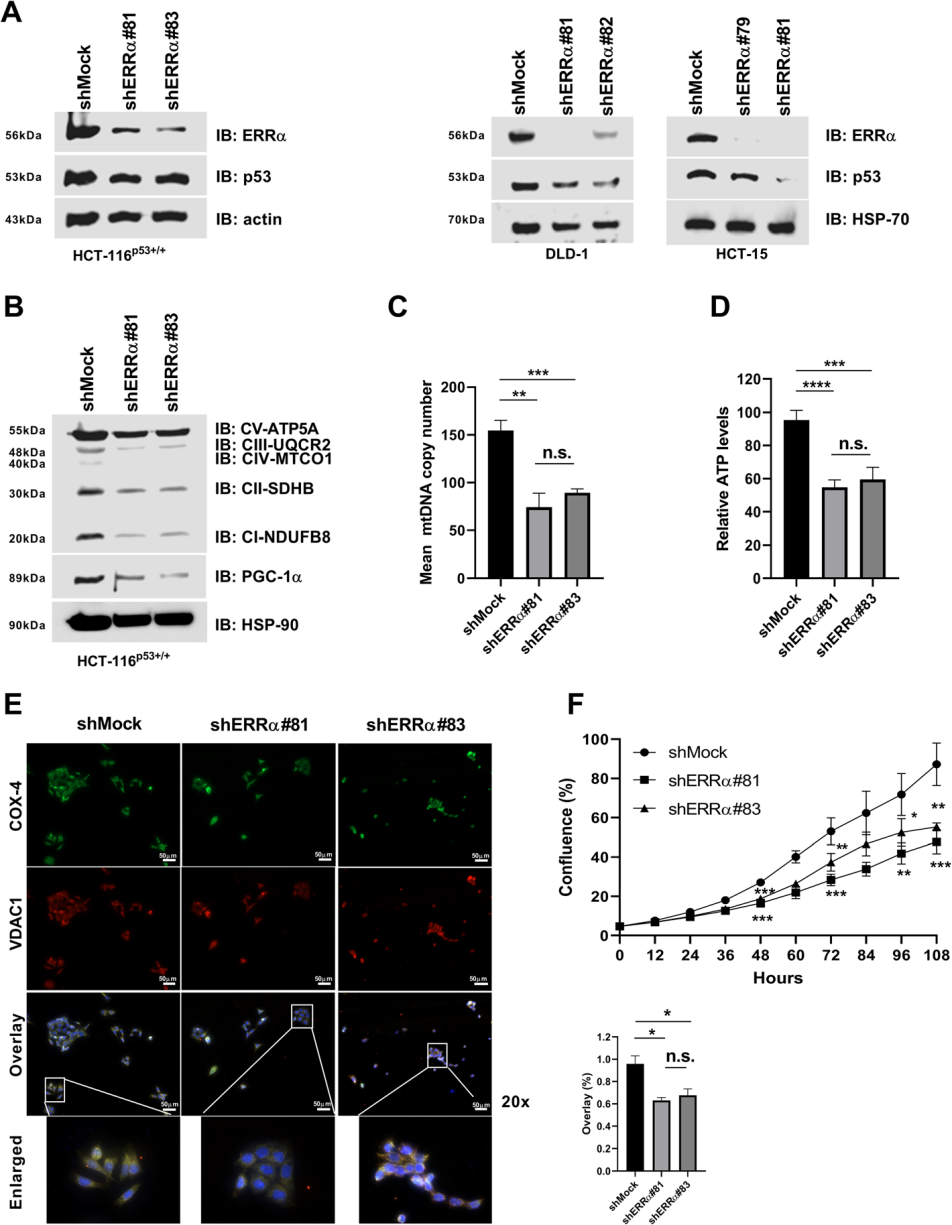
Gene silencing of ERRα affects p53 biology leading to mitochondrial dysfunction and less proliferation. **a** HCT-116^p53+/+^ (*left*), DLD-1 (*middle*), and HCT-15 (*right*). IB analysis was conducted using anti-ERRα, anti-p53, anti-actin, and anti-HSP-70. **b** IB analysis was conducted in HCT-116^p53+/+^ cells using the anti-total OxPhos WB antibody cocktail, anti-PGC-1α, or HSP-90. **c**, **d** Mitochondrial DNA copy number analysis and the intracellular ATP levels were measured in HCT-116^53+/+^ cells as described in the “Methods” section. **e** Immunofluorescence (IF) analysis for COX-4 and VDAC1 was conducted in HCT-116^53+/+^ cells. Panels represent selected digitally enlarged portions of parent images to enhance the visibility of COX-4 and VDAC1. Co-localization of COX-4 and VDAC1 was quantified (as % overlay). Scale bar, 50 μm. **f** HCT-116^p53+/+^ cell growth was analyzed. All cells were stably transduced with lentiviral constructs expressing an shRNA specific to ERRα (shERRα#) or an shRNA non-targeting construct (shMock). The data are shown as means ± S.D. (*n* = 2–4). The *p* value was calculated using a two-tailed Student’s *t* test. *p ≤ 0.05; **p ≤ 0.01; ***p ≤ 0.001; ****p ≤ 0.0001; n.s., not significant

As indicated earlier, *TP53* remains the most elusive target in cancer [34, 52], whereas ERRα can be potentially inhibited by drugs [53–55]. Therefore, to unravel the function and to understand the role of the ERRα and p53 complex in colon cancer, we first used short hairpin RNA against ERRα in different colon cancer cell lines to knock down ERRα protein expression. Knocking down ERRα in HCT-116^p53+/+^ (p53 wild-type), DLD-1, or HCT-15 (missense or GOF p53 mutants) colon cancer cell lines reduced the expression of the p53 protein, independent of p53 status (Fig. 2a). Because ERRα and p53 each govern a wide array of mitochondrial network genes [26, 41], we investigated whether the inhibition of ERRα might affect mtOxPhos activity. We measured the protein levels of the mtOxPhos complex subunits I-V (ATP5A, UQCR2, MTCO1, SDHB, and NDUFB8) in HCT-116^p53+/+^ cells and also determined the expression of PGC-1α, a key regulator of mtOxPhos activity and mitochondrial biogenesis, in these cells. Results showed a profound decrease in both mtOxPhos and PGC1α protein expression after knocking down ERRα (Fig. 2b). Furthermore, gene silencing of *ERRα* in HCT-116^p53+/+^ cells significantly decreased the mitochondrial DNA (mtDNA) copy number and the relative levels of intracellular ATP (Fig. 2c, d). We next used IF to determine mitochondrial biogenesis through two mitochondrial markers, the cytochrome *c* oxidase subunit IV (COX-4) and the voltage-dependent anion channel 1 (VDAC1). We observed a significant reduction of COX-4 and VDAC1 expression in HCT-116^p53+/+^ cells after knocking down ERRα (Fig. 2e). Interestingly, gene silencing of *ERRα* in HCT-116^p53−/−^ (additional file Fig. S3A) showed a substantial change in the distribution of the co-localization of both mitochondrial markers from an elongated (enlarged shMock) to a fragmented network (enlarged shERRα#81 and shERRα#83) and a significant difference in the expression of COX-4 and VDAC1 when compared with HCT-116^p53+/+^ cells (additional file Fig. S3B). Finally, we observed less cell proliferation and cell cycle arrest in response to knocking down ERRα regardless of the status of p53 (Fig. 2f and additional file Fig. S3C-E). For the first time, these results provide evidence showing that gene silencing of *ERRα* affected p53 expression, regardless of p53 status. Targeting ERRα affected cell proliferation through the regulation of mitochondrial function and biogenesis independently of p53 status (i.e., whether p53 wild-type or deficient).

### Changes in the mitochondrial proteomic profile imposed by the pharmacological inhibition of ERRα

**Fig. 3:**
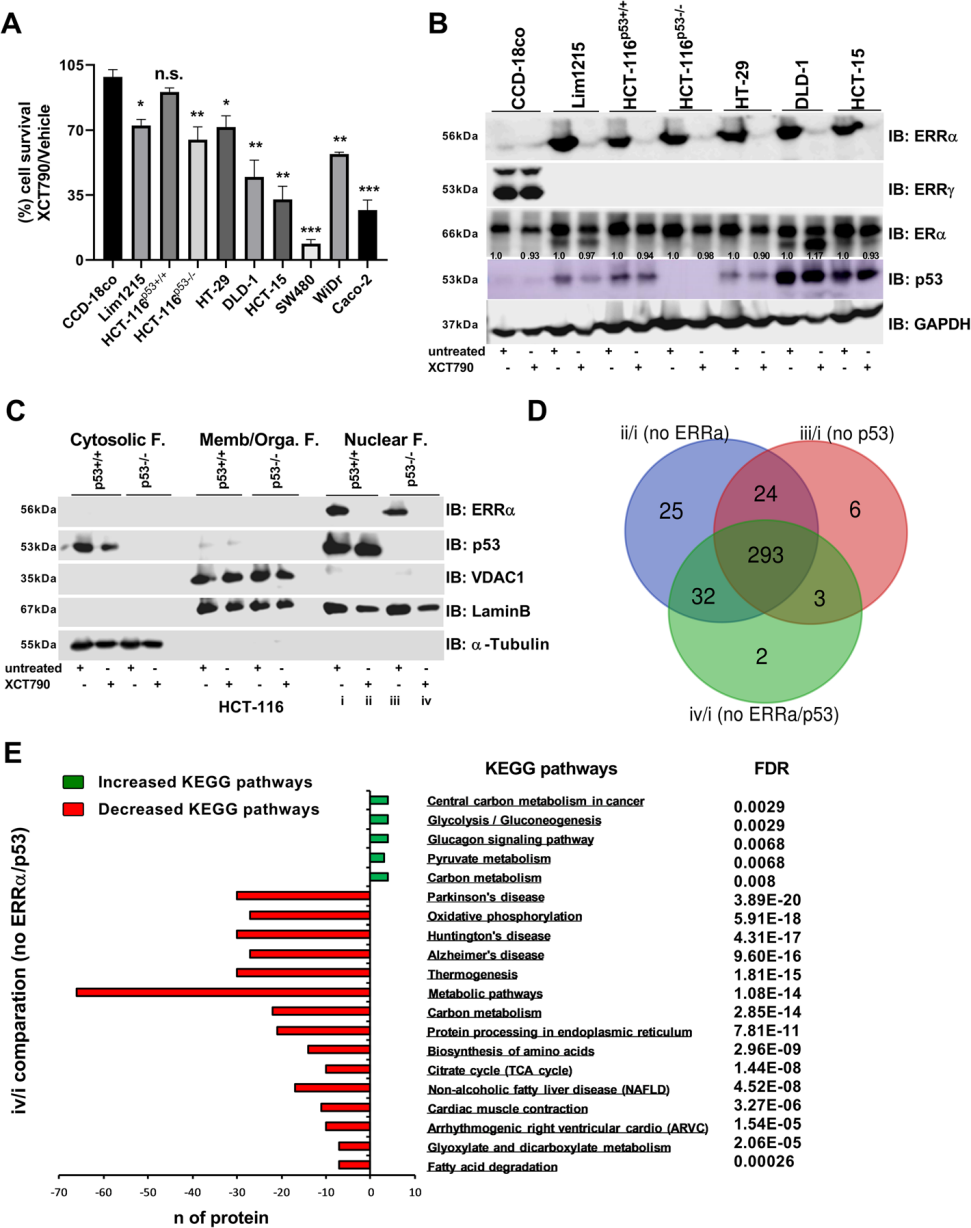
The ERRα and p53 complex promotes mitochondrial function. **a** Cell viability was assessed by using crystal violet staining in CCD-18Co colon fibroblasts and a panel of colon cancer cell lines (Lim1215, HCT-116^p53+/+^, HCT-116^p53−/−^, HT-29, DLD-1, HCT-15, SW480, WiDr, and Caco-2). **b** IB analysis was performed using anti-ERRα, anti-ERRγ, anti-ERα, anti-p53 (low and high membrane exposition), and anti-GAPDH in CCD-18Co colon fibroblasts and a panel of colon cancer cell lines (Lim1215, HCT-116^p53+/+^, HCT-116^p53−/−^, HT-29, DLD-1, and HCT-15). **c** The cytosolic, membrane/organelle, and nuclear fractions were purified from XCT790-treated and untreated HCT-116^p53+/+^ and HCT-116^p53−/−^ cells, and then IB analysis was performed using anti-ERRα, anti-p53, anti-VDAC1, anti-LaminB, and anti-α-Tubulin. **d** Venn diagram analysis examining the proteomic profile of the membrane/organelle fraction within three different comparisons: ii/i (no ERRα), iii/i (no p53), and iv/i (no ERRα/p53) as identified by mass spectrometry (*n* = 3 biological replicates per group). The bioinformatics tool found at http://bioinformatics.psb.ugent.be/webtools/Venn/ was used to generate the Venn diagram analysis. **e** Enriched KEGG pathways upregulated and downregulated obtained by STRING analysis of the membrane/organelle fraction comparing (iv) the absence of ERRα and p53 with the (i) presence of ERRα and p53. Comparisons between groups were made using multiple *t* tests with a false discovery rate of *p* ≤ 0.01. All cells were treated for 48 h with XCT790 (15 μM) or vehicle (DMSO). The data are shown as means ± S.D. (*n* = 3). The *p* value was calculated using a two-tailed Student’s *t* test. *p ≤ 0.05; **p ≤ 0.01; ***p ≤ 0.001; n.s., not significant

Several antagonists have been designed that lead to the degradation of ERRα [55, 56]. However, whether ERRα antagonists can block the interaction between ERRα and p53 is unknown. Thus, to extend our analysis of the effectiveness of targeting the direct binding of ERRα and p53 in colon cancer, we selected an antagonist of ERRα known as XCT790. First, to determine whether or not XCT790 might be a specific antagonist of ERRα expression in colon cancer cell lines [57, 58], we used CCD-18co normal colon fibroblasts and a panel of nine different colon cancer cell lines, including colon cancer cells expressing wild-type p53 (HCT-116^p53+/+^ and Lim1215), p53 mutant (missense or GOF; HT-29, DLD-1, HCT-15, SW480, and WiDr), and p53-deficient (HCT-116^p53−/−^ and Caco-2) cells. XCT790 treatment decreased proliferation in almost all colon cancer cell types compared to normal colon cells (Fig. 3a). We extended our analysis by performing an IB analysis with ERRα, ERRγ, and ERα in normal CCD-18co colon fibroblasts and in a panel of colon cancer cell lines (Lim1215, HCT-116^p53+/+^, HCT-116^p53−/−^, HT-29, DLD-1, and HCT-15). Results showed that treatment of cells for 48 h with XCT790 (up to a concentration of 15 μM) totally abrogated the protein expression level of ERRα and slightly affected the expression level of ERα (Fig. 3b). Interestingly, the ERRα protein seemed to be highly expressed in colon cancer cells compared to normal colon fibroblasts and was inversely associated with the expression of ERRγ, which was not expressed in colon cancer cells (Fig. 3b). Similar to knocking down ERRα expression, the treatment of cells with XCT790 decreased p53 protein expression in most colon cancer cell lines, independently of p53 status (Fig. 3b). Notably, using a transient transfection system to overexpress ERRα restored the expression of p53 to normal levels in HCT-116^p53+/+^ cells (additional file Fig. S4A).

Despite the orchestration of mitochondrial bioenergetics of tumors by ERRα, no scientific evidence exists underlying the mechanism by which ERRα could be therapeutically exploited as a new metabolic vulnerability in the presence or loss of wild-type p53 function. We next sought to delineate this mechanism by using quantitative proteomics analysis by SWATH-MS in the mitochondrial subcellular membrane/organelle fractionation (Fig. 3c). We compared the mitochondrial proteomics profile of four different cellular conditions, including (i) HCT-116^p53+/+^ cells with intact ERRα and p53 function; (ii) HCT-116^p53+/+^ cells with intact p53 function, but with suppressed ERRα expression; (iii) HCT-116^p53−/−^ cells with intact ERRα function, but no p53 function; and (iv) HCT-116^p53−/−^ cells with suppressed ERRα expression and no p53 function (Fig. 3c). A Venn diagram was used to show a comparison of the mitochondrial proteomic profile of 3 different combinations, including no ERRα (ii/i), no p53 (iii/i), and no ERRα and no p53 (iv/i). The results showed a total of 293 identical mitochondrial proteins (76% of the proteins analyzed) within the three groups that exhibited decreased expression levels (Fig. 3d, Table S1). A comparison of cells without ERRα and p53 (iv) with cells expressing the ERRα and p53 complex (i) showed that the absence of the ERRα/p53 complex affected central metabolism by altering KEGG pathways (Kyoto Encyclopedia of Genes and Genomes). The increased activation of KEGG pathways is related to central carbon metabolism in cancer and glycolysis, and profound decreases in the activation of KEGG pathways is associated with Parkinson’s disease, OxPhos, Huntington’s disease, Alzheimer’s disease, thermogenesis, and several metabolic pathways (false discovery rate ≤ 0.01; Fig. 3e, Table S1). Intriguingly, comparing the absence of ERRα only (ii) or the absence of p53 only (iii) to the presence of the ERRα and p53 complex (i) indicated that the absence of the function of a single protein (ERRα or p53) affected central metabolism, such as glycolysis and carbon metabolism, by increasing the activation of KEGG pathways, and dramatically decreased the activation of KEGG pathways associated with mitochondrial metabolism, such as Parkinson’s disease and OxPhos (FDR ≤ 0.05; additional file Fig. S4B, C, Table S1). Overall, our results showed that ERRα is highly expressed in colon cancer and directly targeting ERRα with XCT790 affected p53 expression. Collectively, these results prompted us to conclude that ERRα and p53 cooperatively control mitochondrial function; however, we could not distinguish whether the effect of ERRα and p53 on mitochondrial function is additive or an independent molecular event.

### Pharmacological inhibition of ERRα impairs mitochondrial biogenesis and mtOxPhos in colon cancer cells

**Fig. 4:**
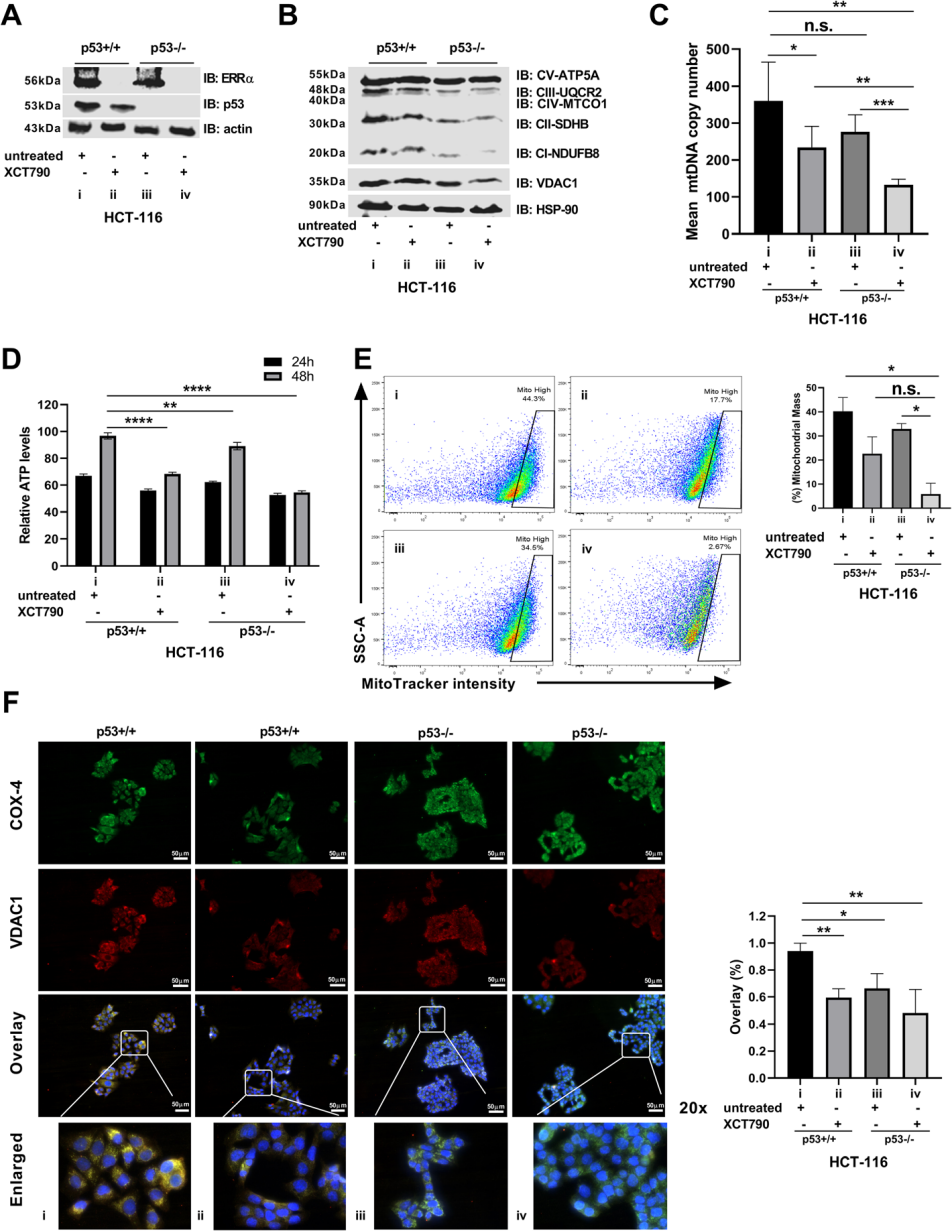
Targeting ERRα affects mitochondrial biogenesis and function dependent on p53. **a** IB analysis was performed using anti-ERRα, anti-p53, and anti-actin. **b** IB analysis was conducted using the anti-total OxPhos WB antibody cocktail, anti-VDAC1, and anti-HSP-90. **c**, **d** Mitochondrial DNA copy number analysis and the intracellular ATP levels were measured as described in the “Methods” section. **e** Cells were stained with MitoTracker Red CMXRos and DAPI and analyzed by flow cytometry. The percentage of the mitochondrial mass (mitoHigh) was quantified. **f** IF analysis to detect COX-4 and VDAC1 was conducted. Enlarged panels represent selected digitally enlarged portions of parent images to enhance the visibility of COX-4 and VDAC1. Co-localization of COX-4 and VDAC1 was quantified (as % overlay). Scale bar, 50 μm. All experiments were conducted using HCT-116^p53+/+^ and HCT-116^p53−/−^ cells treated for 48 h with XCT790 (15 μM) or vehicle (DMSO). Data are shown as means ± S.D. (*n* = 2–3). The *p* value was calculated using a two-tailed Student’s *t* test. *p ≤ 0.05; **p ≤ 0.01; ***p ≤ 0.001; ****p ≤ 0.0001; n.s., not significant

To further determine the molecular mechanisms underlying the function of the ERRα and p53 complex in cooperatively promoting colon cancer survival through the regulation of mitochondrial biogenesis and mtOxPhos, we analyzed the effect of the presence or absence of ERRα and p53 (Fig. 4a). We found that the absence of the ERRα and p53 complex (iv), but not the absence of either ERRα (ii) or p53 (iii) only, gradually decreased the protein levels of all five mtOxPhos complex subunits (complex I-V) and VDAC1 (Fig. 4b). Indeed, in HCT-116^p53−/−^ cells, the expression of mtOxPhos markers and VDAC1 was strongly downregulated following treatment with XCT790 (Fig. 4b). To gain more insight into the mechanism of the ERRα and p53 complex in coordinating mitochondrial function, we measured the mtDNA copy number and the relative levels of intracellular ATP. Consistent with the results above, the absence of the ERRα and p53 complex (iv), but not the absence of either ERRα (ii) or p53 (iii) only gradually decreased the mtDNA copy number and the relative levels of intracellular ATP (Fig. 4c, d). Again, HCT-116^p53−/−^ cells showed the most dramatic decrease in mtDNA copy number and lower levels of intracellular ATP in a time-dependent manner following treatment with XCT790 (Fig. 4c, d). Subsequently, we measured mitochondrial mass using MitoTracker Red CMXRos. Again, treated HCT-116^p53−/−^ cells (iv) showed a significant decrease in the mitochondrial mass compared to untreated HCT-116^p53−/−^ cells (iii) or HCT-116^p53+/+^ control cells (i), but not HCT-116^p53+/+^ cells treated with XCT790 (ii) (Fig. 4e).

Next, we used IF to quantify mitochondrial biogenesis by using two mitochondrial markers, COX-IV and VDAC1. We observed a reduction in the co-localization of COX-IV and VDAC1 in HCT-116^p53+/+^ cells treated with XCT790 (ii), untreated HCT-116^p53−/−^ cells (iii), and treated HCT-116^p53−/−^ cells (iv) compared to the presence of the complex (i) (Fig. 4f). Also, we observed changes in the distribution of the co-localization of both mitochondrial markers from an elongated (enlarged) to a fragmented network, which was observed in the absence of the ERRα and p53 complex (Fig. 4f). Thus, one might reasonably expect that affecting mitochondrial biogenesis causes mitochondrial metabolic stress leading to accumulation of mitochondrial abnormalities, such as mitochondrial dysfunction, ROS production, and MMP [59].

### Targeting ERRα causes mitochondrial metabolic stress leading to more cytotoxicity in colon cancer with loss of wild-type p53 function

**Fig. 5:**
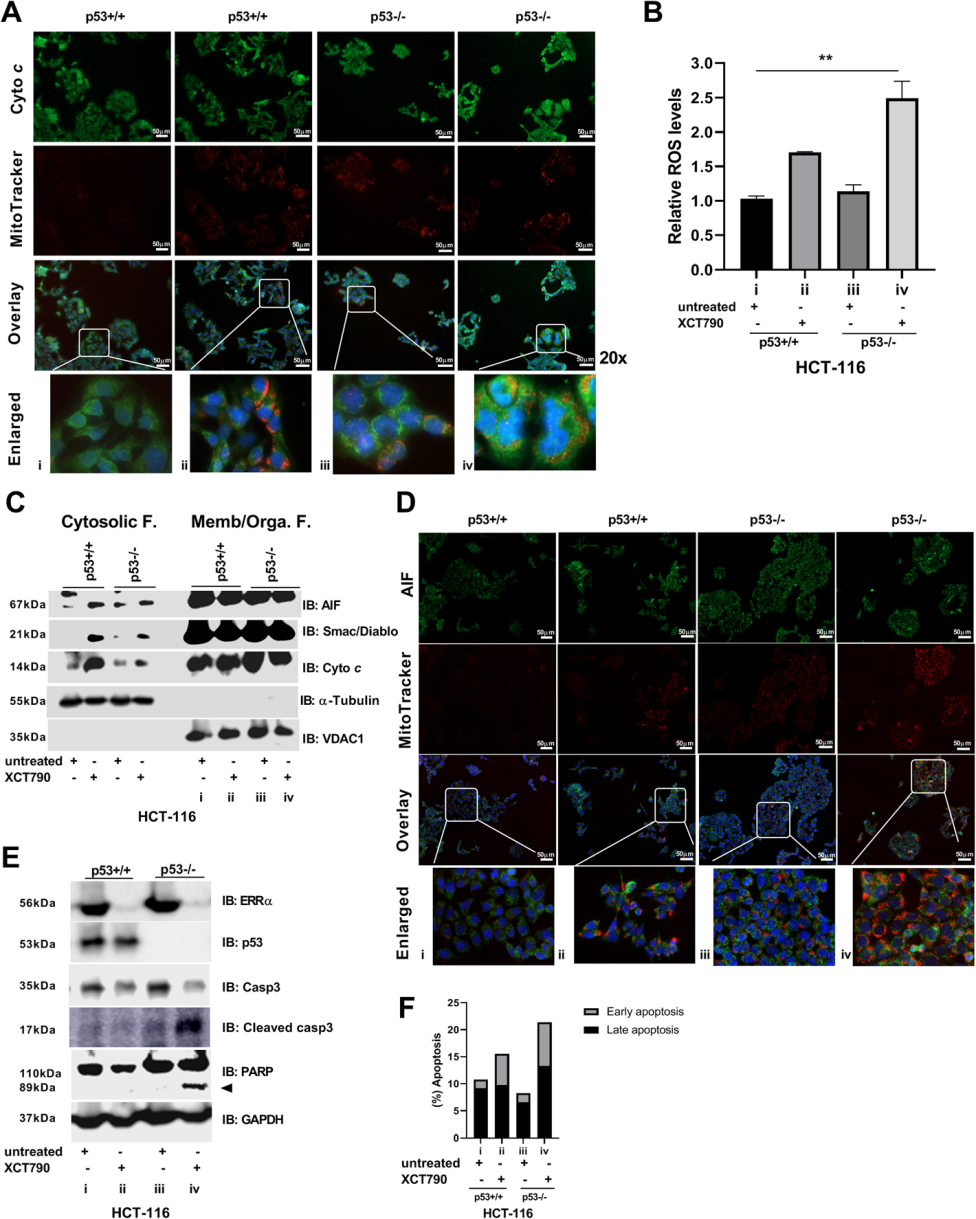
Targeting ERRα increases mitochondrial dysfunction leading to apoptosis dependent on the presence of p53. **a** Live cells were stained with MitoTracker Red CMXRos, and IF analysis was conducted to detect cytochrome *c* (cyto *c*). Enlarged panels represent selected digitally enlarged portions of parent images to enhance the visibility of mitochondrial ROS (orange dots); scale bar, 50 μm. **b** Cells were stained with H_2_DCFDA and DAPI and analyzed by flow cytometry. ROS levels were determined by the conversion of H_2_DCFDA to the highly fluorescent 2′,7′-dichlorofluorescein (DCF) as described in the “Methods” section. **c** The cytosolic and membrane/organelle fractions were analyzed by IB with anti-AIF, anti-Smac/Diablo, anti-cyto *c*, anti-α-Tubulin, and anti-VDAC1. **d** Live cells were stained with MitoTracker Red CMXRos, and IF analysis was conducted for AIF. Enlarged panels represent selected digitally enlarged portions of parent images to enhance the visibility of mitochondrial ROS (orange dots; scale bar, 50 μm). **e** IB analysis was conducted with anti-ERRα, anti-p53, anti-caspase-3, anti-cleaved caspase-3, anti-cleaved PARP, and anti-GAPDH. **f** Apoptotic cells were detected by flow cytometry and by using the Annexin V-FITC assay kit as described in “Methods” section. All experiments were performed using HCT-116^p53+/+^ and HCT-116^p53−/−^ cells treated for 48 h with XCT790 (15 μM) or vehicle (DMSO). Data are shown as means ± S.D. (*n* = 2–3). The *p* value was calculated using a two-tailed Student’s *t* test. **p ≤ 0.01

We stained live cells first with MitoTracker Red CMXRos and with cytochrome *c* (cyto *c*). By using IF microscopy, we observed that the absence of the ERRα and p53 complex (iv), but not the absence of either ERRα (ii) or p53 (iii) only, gradually impacted the increased level of oxidized mitotracker (orange spots) and diffused cyto *c* across the cytosol and nucleus (Fig. 5a). We also measured the levels of intracellular ROS and the presence of mitochondrial-apoptotic proteins in the cytosol. As expected, in response to ERRα inhibition followed by treatment with XCT790 in HCT-116^p53−/−^ cells (iv), we observed significantly increased levels of ROS (Fig. 5b). Moreover, in the absence of the ERRα and p53 complex (iv) and also in the absence of only ERRα (ii) or p53 (iii), we observed a leaking from mitochondria to cytosol of apoptosis-inducing factor (AIF), Smac/Diablo, and cyto *c* (Fig. 5c). To confirm these results, we stained live cells with MitoTracker Red CMXRos and then with an AIF antibody. By using IF microscopy, we observed that the absence of either ERRα (ii) or p53 (iii) only gradually increased the level of oxidized mitotracker (orange dots) and diffused AIF into the nucleus (Fig. 5d). Notably, XCT790 caused a marked G1 cell cycle arrest and less proliferation regardless of p53 status (additional file Fig. S5A-C). However, the absence of the ERRα and p53 complex (iv) showed increased cleaved forms of two apoptotic markers, caspase-3 and poly (ADP-ribose) polymerase (PARP), and more accumulation of Annexin V apoptotic cell staining leading to an irreversible apoptotic signaling cascade (Fig. 5e, f). Overall, these results provided evidence that loss of the ERRα and p53 complex progressively affected mitochondrial biogenesis, mtOxPhos, ROS production, and MMP, leading to more mitochondrial metabolic stress and apoptosis.

### XCT-790 impairs the growth of p53-deficient cells and of p53 mutant patient-derived colon xenografts

**Fig. 6:**
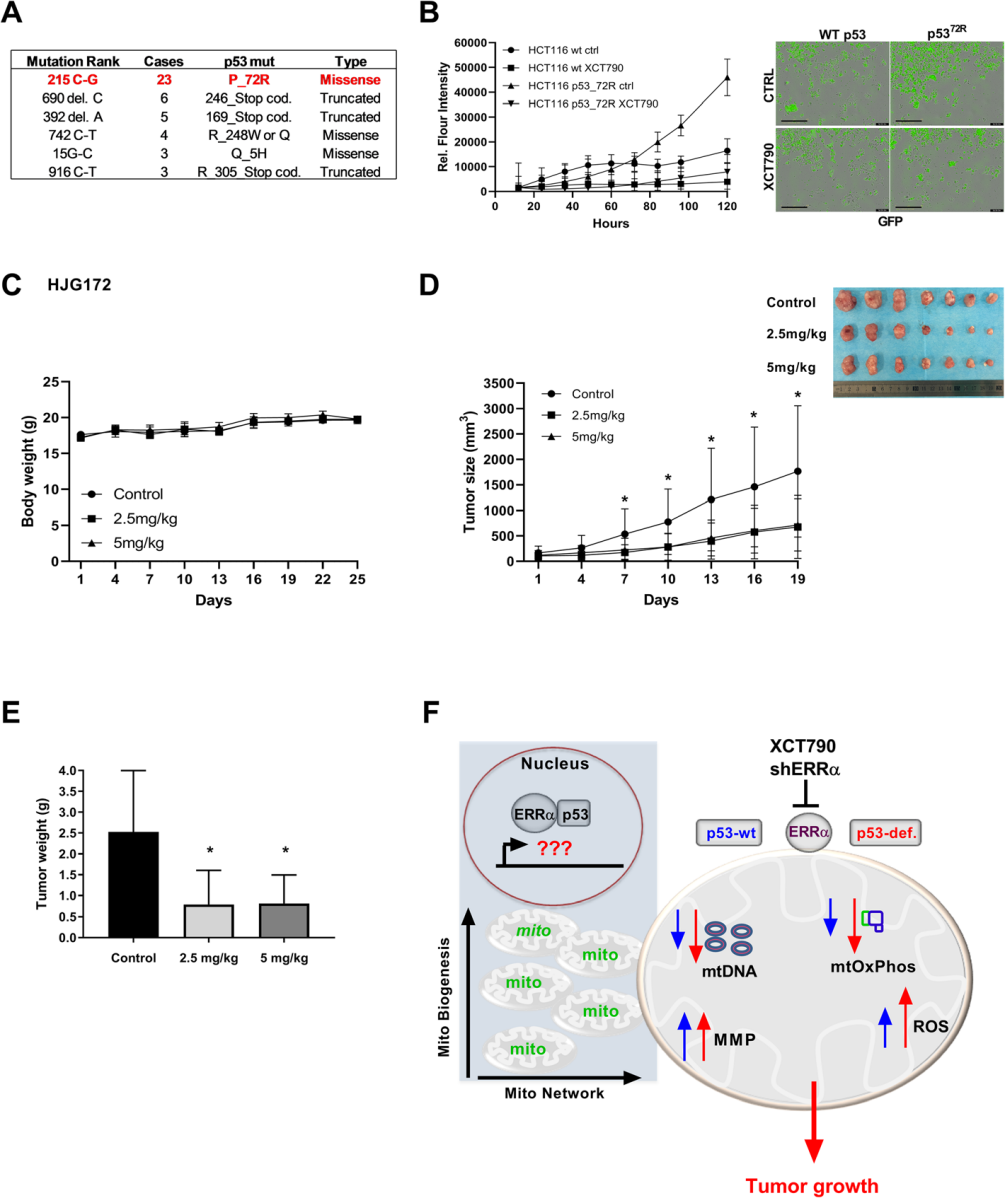
XCT790 attenuates the growth of PDX tumors in mice. **a** Direct DNA sequencing of the complete p53 genomic locus was performed for 37 samples of PDX colon cancers. Highlighted in the box are the point mutation detected in p53 and the number of mutated patient samples. **b** HCT-116^p53+/+^ and HCT-116^p53_72R^ cell growth was analyzed. HCT-116^p53_72R^ cells were stably transduced with lentiviral constructs expressing mutant p53^72R^ fused to EGFP/luciferase, and HCT-116^p53+/+^ cells were stably transduced with lentiviral control construct expressing EGFP/luciferase. HCT-116^p53+/+^ and HCT-116^p53−/−^ cells were treated with XCT790 (15 μM) or vehicle (DMSO). **c** Body weight of mice was plotted over 25 days. **d** Growth of HJG172 PDX tumors was monitored for the entire duration of the experiment. Data represent means ± SEM. An unpaired *t* test was used to determine statistical significance; *P < 0.05. Images show the tumor xenografts dissected at the end of the experiments. **e** At the end of the treatment period, all animals were euthanized, and tumors were extracted and weighed. Data represent mean ± SEM. An unpaired *t* test was used to determine statistical significance; *P < 0.05. **f** Proposed model illustrating how the ERRα/p53 complex modulates mitochondrial biogenesis and function in colon cancer. Targeting ERRα in p53-deficient cells decreased the mtDNA copy number and mtOxPhos activity leading to an increased production of mitochondrial and intracellular ROS. XCT790 might be considered a potential drug to treat colon cancer patients

To understand the clinical relevance of targeting ERRα in wild-type p53, p53 mutant (missense or GOF), and p53-deficient colon cancer patient samples, we performed direct DNA sequencing of the complete p53 genomic locus of 37 samples that came from patient-derived colon tumors. Overall, we identified 33 mutant p53 tumors and 4 wild-type p53 tumors (additional file Fig. 6a). Comparing the mutant p53 tumors, we identified 18 cases of nonsense mutant p53 tumors (p53-deficient) and 15 cases of missense mutant p53 tumors (GOF). Intriguingly, the single nucleotide polymorphism (SNP) 215C > G that translates to a P72R codon mutation was found in 63% of the patient cohort (Fig. 6a). Based on these data, we created matched HCT-116^p53−/−^ (p53-null background) cells containing the tumor-derived mutant form of p53 P72R fused with GFP and luciferase, along with the vector-transfected control HCT-116^p53+/+^ parental cell line (additional file Fig. S6B). Colon cancer cell lines, HCT-116^p53+/+^ (wild-type p53) and HCT-116^p53_P72R^, were subjected to treatment with XCT790 (15 μM) to examine proliferation. The results indicate that mutant HCT-116^p53_P72R^ cells showed significantly increased proliferation compared with wild-type p53-expressing cells, and treatment with XCT790 was very effective at inhibiting growth of both wild-type and mutant p53-expressing cells (Fig. 6b).

To illustrate the impact of the genetic status of p53 on the efficacy of XCT-790, we used an in vivo tumor model with patient-derived colon tumor explants grown in SCID mice. We selected and established a missense mutant p53 genotype (single mutant P72R: HJG172, additional file Fig. 6a). DNA sequencing showed that HJG172 contains a single point mutation (215C > G), the most frequent in the patients analyzed (Fig. 6a). Mice bearing xenografts were treated with vehicle or one of two different concentrations of XCT790 (2.5 mg/kg or 5 mg/kg body weight) every 3 days over a 21-day period.

Notably, a preliminary random toxicity study was performed, showing that neither XCT790 concentration significantly affected liver or spleen weight in mice (*n* = 4; additional file Fig. S6C). As expected, neither concentration of XCT790 affected body weight, liver, or spleen weights of HJG172 (mut ^p53_72R^; Fig. 6c and additional file Fig. S6D). Interestingly, we observed a significant reduction in tumor growth and tumor weight of HJG172 (mut ^p53_72R^), compared to vehicle-treated control tumors, with a delay of ~ 40% tumor growth (Fig. 6d, e). Taken together, these results provide evidence that XCT790 might be considered as a potential drug to treat colon cancer patients with defective p53 status (Fig. 6f).

## Discussion

The tumor metabolic phenotype is controlled by intrinsic genetic mutations, such as loss of p53 function and external responses, including hypoxia, pH, and nutrient availability [60]. These altered signals move central metabolism toward a cascade of events capable of reprogramming cellular metabolism to match the requirement for cell growth and proliferation [61]. The maintenance of proper mitochondrial function seems to be essential to maintain the bioenergenetic capacity of cells, provide anabolic substrates, control redox and calcium homeostasis, participate in transcriptional regulation, and govern oncogenesis [62]. Various TFs and NRs regulate mitochondrial biogenesis and function [63]. Notably, the TF p53 and the NR ERRα both play potential roles in regulating gene expression of mitochondrial pathways [25, 26, 41, 63]. However, little is known about whether ERRα and p53 directly interact and potentially coordinate mitochondrial pathways toward tumor survival. We are the first to report that the ERRα LBD directly interacts with the p53 CTD, independent of p53 protein mutational status. ERRα and p53 cooperate in the promotion of mitochondrial biogenesis and mtOxPhos. Therefore, targeting ERRα activates mitochondrial metabolic stress that in turn induces colon cancer cell cycle arrest and apoptosis, which is dependent on the presence of p53. Here we propose the use of XCT790 as a single agent to target ERRα resulting in acute metabolic stress and cytotoxicity mediated through the p53 pathway and dependent on p53 status (Fig. 6f).

The *TP53* gene is known to be the most frequent target for mutation in human cancers [34]. The *TP53* mutational spectrum in human cancer is highly concentrated in the DNA-binding domain (DBD) of the p53 protein (100 to 306 amino acid residues) with the vast majority of cancer-associated mutations being missense mutations or GOF [35, 64]. Interestingly, our data provide unequivocal evidence showing that regardless of p53 status, whether wild-type p53 or a missense p53 mutant, the interaction between ERRα and p53 exists in colon cancer. Consistent with our experimental data, we created a computational model that predicted three potential protein-protein interfaces between the ERRα LBD and the p53 CTD. These protein-protein interfaces can be further exploited to understand the underlying structural biology of the complex. Moreover, our study answered an important question raised by Kim et al., where they commented on whether proteins that could partner with p53 mutant proteins are essential for GOF function and whether the binding differs based on the site of the missense mutation [64].

Because the interaction of the ERRα/p53 complex is independent of p53 mutation status, a rational strategy for therapeutically targeting ERRα in colon cancer needs to be developed. We found that silencing *ERRα* or pharmacological inhibition of ERRα expression independently of p53 status directly affected p53 expression and p53-dependent transcriptional programming, including p21^WAF1/CIP1^ and cyclin D1, which led to cell cycle arrest and decreased proliferation. One likely explanation for this observation is that the ERRα/p53 complex may cooperatively control mitochondrial biogenesis and function leading to increased cell survival. In support of this possibility, knocking down or pharmacologically inhibiting the expression of ERRα clearly affected mtOxPhos and mitochondrial biogenesis in colon cancer.

As indicated above, we considered targeting ERRα rather than p53 because ERRα is a druggable protein [16, 28, 63], and the interaction between ERRα and p53 still exists regardless of the mutational status of p53. Several studies showed that targeting ERRα significantly decreased the respiratory capacity of melanoma cells and mitochondrial function in breast cancer [12, 28]. However, whether targeting ERRα could have some advantage depending on p53 status was unclear. In our study, we used XCT790, a specific antagonist of ERRα with the capacity to interact with ERRα and disrupt the interaction of the ERRα/PGC1α complex and its expression [58]. The ERRα/PGC1α complex has been reported to regulate mitochondrial biogenesis [65], and XCT790 disrupted this complex interaction with growth-inhibitory therapeutic effects in breast cancer [12]. Several groups have developed antagonists of the ERRα/PGC1α complex, including XCT790 and compounds (Cpd) A, 1a, and 29 [54, 55, 66]. However, no other evidence has been provided to determine whether XCT790 could disrupt the interaction of the ERRα/p53 complex or its expression. We found that XCT790 decreased the expression of ERRα but did not significantly affect the expression of ERRγ or ERα. Importantly, the pharmacological inhibition of ERRα recapitulated the results obtained with a gene silencing approach, which prompted us to better understand the function of the ERRα/p53 complex.

Based on these data, we used isogenic colon cancer cells HCT-116 wild-type p53 and p53-null background to study the absence and presence of the ERRα/p53 complex. In the absence of the complex, the proteomic profiling by KEGG pathways revealed an increase in components of glucose metabolism and a substantial decrease in components of mtOxPhos. One likely explanation is that ERRα and p53 together control mitochondrial function. However, the absence of a single protein or ERRα function (ii/i) or p53 function (iii/i) is sufficient to significantly affect components of mitochondrial function. Therefore, possibly targeting mitochondrial as a metabolic vulnerability will render cells more susceptible to death is feasible.

Cellular reliance on mitochondrial metabolism is associated with drug resistance and correlates with poor patient outcome in many cancers [14]. For example, the ERRα/PGC-1α transcriptional axis is considered to be a major regulator of mitochondrial metabolism mediating metabolic adaptations driving drug resistance in breast cancer and melanoma [28, 67–69]. In fact, several groups have shown that disruption of the mitochondrial network can lead to metabolic stress and cell death [16, 59], which confirms the large numbers of ongoing clinical trials assessing the efficacy of potential mitochondrial metabolic blockers [70, 71]. However, little is known about the effect that the ERRα/p53 complex cooperatively exerts on mitochondrial function and whether it can be accessed for clinical intervention in colon cancer. Notably, the inhibition of ERRα expression showed more efficacy in the absence of p53 function, revealing that targeting ERRα in the absence of p53 is more effective to drive mitochondrial metabolic stress, such as production of mitochondrial and intracellular ROS. A previous report showed that the general mitochondrial uncoupling drug, niclosamine, might be used as anticancer therapy against p53-defective cancers [44]. Herein, we confirmed the role of the ERRα/p53 complex, which is required to maintain mitochondrial function in colon cancer and targeting ERRα resulted in mitochondrial dysfunction, causing higher cytotoxicity in cells with loss of p53. Together, our data showed for the first time the interdependence of the ERRα and p53 complex in cooperatively promoting colon tumor survival through the regulation of mitochondrial function. Our data also suggested that targeting ERRα in p53-deficient tumors might have a larger effect in killing colon cancer cells. Importantly, the efficacy of XCT790 against the p53-defective pathway is recapitulated in in vivo tumor models, showing increased anti-tumorigenic effects on p53-mutant tumors in a colon patient-derived xenograft model (Fig. 6).

This suggests that the ERRα/p53 complex can be exploited as a metabolic vulnerability rendering the presence or absence of the p53 protein as a selective criterion for future clinical approaches. Because several challenges exist in directly targeting p53, ERRα might be considered a potential therapeutic target for colon cancer.

## Conclusions

Here, we identified the interaction of ERRα and p53 and, as discussed above, we have elucidated, at least in part, the cellular mechanisms of the ERRα/p53 complex, which is required to maintain mitochondrial function in colon cancer. The PDX model of colon cancer, established for p53-deficient cells, showed a significant reduction in tumor growth and weight in mice with an approximate 40% delay in tumor growth, when treated with XCT790 compared to vehicle-treated control tumors. Our findings suggest the possibility of targeting colon cancers defective in p53 function by using XCT790 or similar molecules. Future studies with a larger sample size, including tumors with different p53 status, are needed to confirm our findings.

## Methods

### Cell culture

The 293T cells were purchased from American Type Culture Collection (Manassas, VA, USA) and maintained in high glucose Dulbecco’s modified Eagle’s medium (DMEM, Invitrogen, Camarillo, CA, USA) supplemented with 10% fetal bovine serum (FBS, Gemini Bio-Products, Calabasas, CA, USA), 100 U/mL penicillin, and 100 mg/mL streptomycin. FreeStyle^TM^ 293-F cells were purchased from Thermo Fisher Scientific (Carlsbad, CA, USA) and maintained in FreeStyle 293 Expression Medium. CCD-18Co normal human colon fibroblasts and human colon cancer cell lines, HCT-116^p53+/+^, DLD-1, HCT-15, HT-29, SW480, WiDr, and Caco-2 were purchased from ATCC. HCT-116^p53−/−^ cells were kindly provided by Dr. Bert Vogelstein [72]. Lim1215 cells were obtained from the European Collection of Authenticated Cell Cultures (ECACC). HCT-116^p53+/+^, HCT-116^p53−/−^, and HT-29 cells were grown in McCoy’s 5A Medium (Invitrogen, Camarillo, CA, USA), supplemented with 10% FBS, 100 U/mL penicillin, and 100 mg/mL streptomycin. CCD-18Co, WiDr, and Caco-2 were grown in Minimum Essential Media (MEM, Invitrogen, Camarillo, CA, USA), supplemented with 10% FBS, 100 U/mL penicillin, and 100 mg/mL streptomycin. DLD-1, HCT-15, and Lim1215 cells were grown in RPMI-1640 (Invitrogen, Camarillo, CA, USA) supplemented with 10% FBS, 100 U/mL penicillin, and 100 mg/mL streptomycin. For Lim1215 cells, we added 0.6 μg/ml insulin and 1 μg/ml hydrocortisone. SW480 cells were maintained in HyClone Leibovitz’s L-15 medium (GE Healthcare, Grand Island, NY, USA), and supplemented with 10% FBS, 100 U/mL penicillin, and 100 mg/mL streptomycin. All cells were cultured in a humidified incubator at 37 °C with 5% CO_2_. All the cell lines were cytogenetically tested and authenticated before the cells were frozen. All the cell lines were maintained in culture no longer than 10–15 passages.

### Patients and tumor specimens

Direct DNA sequencing of the complete p53 genomic locus was performed from tumor tissues extracted from human biopsies. DNA was sequenced for the exons of p53 sequence with primers listed at https://p53.iarc.fr/Download/TP53_SangerSequencing_IARC.pdf.

### Animal studies, PDX model

Six- to eight-week-old SCID mice (Vital River Labs, Beijing, China) were used for these experiments. This study was approved by the Ethics Committee of Zhengzhou University (Zhengzhou, Henan, China). The PDX tumor mass was subcutaneously implanted into the back of SCID mice. When tumors reached an average volume of 100 mm^3^, randomized cohort of mice were divided into three groups and subjected to oral gavage with (1) XCT790 at 2.5 mg/kg (*n* = 7); (2) XCT790 at 5.0 mg/kg (*n* = 7); or vehicle control (*n* = 7). XCT790 was administrated every 3 days for 21 days. Tumor volume was calculated from measurements of three diameters of the individual tumor base using the following formula: tumor volume (mm^3^) = (length × width × height × 0.52). Mice were monitored until tumors reached 1.0 cm^3^ total volume, at which time mice were euthanized and tumors extracted.

### Mammalian cell transfection

The plasmid pCMV flag ERRα was purchased from Addgene Inc. (Cambridge, MA, USA). For overexpression of the ERRα protein, the plasmid was transfected into 293T cells by using the iMFectin PolyDNA Transfection Reagent (GenDepot, Barker, TX, USA) following the manufacturer’s suggested protocols. The transfection medium was changed after an overnight transfection and then replaced with fresh growth medium. Cells were cultured for 48 h and then harvested in phosphate-buffered saline (PBS) for further analysis, such as immunoprecipitation (IP) and immunoblotting (IB). For large-scale transient transfection of the *ERRα* gene, the plasmid was transfected into FreeStyle^TM^ 293-F cells by using polyethylenimine (PEI, Sigma-Aldrich, St. Louis, MO, USA) following the manufacturer’s suggested protocols. Cells were cultured for 48 h and then centrifuged at 4 °C for 5 min at 1000 rpm. The cell pellet was used for further analysis, such as protein purification.

### Protein purification

The cell pellet was rinsed in PBS and disrupted in 5 ml lysis buffer (50 mM HEPES, pH 7.8, 300 mM NaCl, 1 mM EDTA, 1 mM DTT, 0.5% (v/v) NP-40, 10% (v/v) glycerol and protease inhibitor cocktail [Sigma-Aldrich, St. Louis, MO, USA]), on ice, for 30 min. The whole cell lysate was centrifuged at 4 °C for 30 min at 15,000 rpm, and the supernatant fraction was incubated at 4 °C on a rotary mixer from 3 to 6 h with ANTI-FLAG®M2 Affinity Gel (Sigma-Aldrich). After incubation, the affinity gel was washed 3 times with wash buffer (50 mM HEPES, pH 7.8, 500 mM NaCl, 1 mM EDTA, 1 mM DTT, 0.02% (v/v) NP-40, 10% (v/v) glycerol and protease inhibitor cocktail [Sigma-Aldrich]). An incubation step was performed with 5 mM ATP and 10 mM MgCl_2_ diluted in wash buffer to remove chaperone protein-binding contamination. Afterwards, the affinity gel was washed 3 times in wash buffer, as described above. The affinity-bound flag-ERRα protein was eluted by adding 5 volumes of elution buffer containing 100 μg/ml of 3X FLAG**®** peptide (Sigma-Aldrich), 50 mM HEPES, pH 7.8, 300 mM NaCl, and 1 mM DTT and left on ice for 1 h each cycle, repeating 4 times. For every cycle, samples were centrifuged at 700×*g* for 3 min, and supernatant fraction containing the eluted protein was collected. The eluted protein was separated by SDS–polyacrylamide gel electrophoresis (PAGE) and stained with Coomassie Blue staining solution (Coomassie R-250, 50% methanol, and 10% acetic acid).

### Identification of ERRα binding partners

To identify endogenous binding partners of ERRα, the purified-ERRα elution fractions were separated by 8% SDS-PAGE and stained with Coomassie stain solution (as above). Proteins in excised gel slices were digested with trypsin (100 ng/sample) and eluted as described [73]. Eluted proteins were digested with Glu-C (100 ng/sample, Promega) for 16 h at 37 °C. LC-MS/MS analysis of enzymatic peptides was performed using Sciex TripleTOF™ 5600 coupled with Eksigent 1D+ nano LC. Raw data were processed and searched with the ProteinPilot™ software (version 4.5) using the Paragon™ algorithm. Protein identification was obtained by searching UniProtKB human database and filtered at ≥ 95% confidence cutoff.

### Size exclusion chromatography

The flag-ERRα purified protein was further purified again using size exclusion chromatography to verify the stability of the complex. The samples were purified on a Superose 6 size exclusion column (GE healthcare, Marlborough, MA, USA) in 50 mM HEPES, pH 7.8, 150 mM NaCl, and 1 mM DTT. The fractions corresponding to ERRα were eluted, collected into a new Eppendorf tube, and loaded into an SDS–PAGE for further (IB) analysis using ERRα and p53 antibodies.

### Immunoprecipitation

Whole cell lysates were prepared with a lysis buffer (137 mM NaCl, 20 mM Tris-HCl, pH 8, 1 mM EDTA, 10% (v/v) glycerol, 1% (v/v) NP-40, and 1 × protease inhibitor tablet). A total of 1000 to 3000 μg of cell lysates were immunoprecipitated with 2 μg of each respective antibody and 40 μL of protein A/G Sepharose beads (Santa Cruz Bio, CA, USA). The samples were rotated at 4 °C overnight. After the beads were washed 3 times with lysis buffer, the pellets were analyzed by SDS–PAGE and transferred to polyvinylidene difluoride (PVDF) membranes. Membrane was blocked with 5% non-fat dry milk for 1 h at room temperature and incubated with the appropriate primary antibody overnight at 4 °C (see Table S2). After 3 washing with PBS containing 0.1% Tween 20, the membrane was incubated with a horseradish peroxidase-conjugated secondary antibody at a 1:5000 dilution, and the signal was detected with a chemiluminescence reagent and the Amersham Imager 600 imager (GE Healthcare, Piscataway, NJ, USA).

### Immunoblot analysis

Cell lysates were prepared with radioimmunoprecipitation (RIPA) assay buffer (50 mM Tris-HCl pH 7.4, 1% (v/v) NP-40, 0.25% (v/v) sodium deoxycholate, 0.1% (v/v) SDS, 150 mM NaCl, 1 mM EDTA, and 1 × protease inhibitor tablet). Equal loading of protein was confirmed by the DC^TM^ protein assay (Bio-Rad, Hercules, CA, USA). Protein sample was separated by SDS–PAGE and transferred to PVDF membranes. Membranes were blocked with 5% non-fat dry milk for 1 h at room temperature and incubated with the appropriate primary antibody overnight at 4 °C (see Table S2). After 3 washes with PBS containing 0.1% (v/v) Tween 20, the membranes were incubated with a horseradish peroxidase-conjugated secondary antibody at a 1:5000 dilution, and the signal was detected with a chemiluminescence reagent using the Amersham Imager 600 imager (GE Healthcare, Piscataway, NJ, USA).

### Proximity ligation assay

To detect the association of ERRα with p53 by PLA, the Duolink in situ red starter kit was used (DUO92101-1KT–Sigma-Aldrich), following the manufacturer protocol exactly as described to perform this study.

### Protein-protein docking of ERRα and p53

The crystal structure of the ERRα LBD in complex with the PGC-1α box3 peptide was downloaded from the Protein Data Bank (PDB; entry 3D24 for ERRα/PGC-1α) and used as a template to build this model of interaction [74, 75]. Next, we found in the scientific literature that the ERα LBD binds directly to p53 [76]. The sequence identity (32.3%) and similarity (56.5%) between the LBDs of ERRα and ERα is in agreement with the highly conserved 3D structure of this domain by superposition (PDB IDs, 3D24 chain A, and 2IOG chain A). On the other hand, the NMR structure (PDB ID: 1DT7, chain X) of the p53 CTD (Lys370-Asp393) shows 22.7% identity and 40.9% similarity with PGC1α box 3 peptide. Thus, the p53 C-terminal regulatory helical domain (PDB ID: 1DT7, chain X) was superposed on PGC1α box 3 peptide in the template (PDB ID: 3D24) followed by removal of PGC1α box 3 peptide and energy minimization. As shown in additional file 2: Figure S2A-H, we developed the model interaction between the ERRα LBD and the p53 CTD. In this model, three potential interaction sites have been identified and the electrostatic surface representation of p53 binding site on ERRα. The 3-D First Fourier Transform–based protein docking algorithm of HEX 8.00 [77] was then used for docking experiments to determine the possible binding mode between ERRα and p53.

### Construction of expression vectors

To construct the V5-His-tagged expression vector of ERRα, the Flag-tagged expression vector of p53, and the EGFP-luciferase-tagged expression vector of mutant p53^P72R^, the DNA sequences corresponding to ERRα LBD (aa 193–423), p53 CTD (aa 300–393), and p53^P72R^ were amplified by PCR. The PrimeSTAR® GXL DNA Polymerase high fidelity enzyme (Takara Bio, Mountain View, CA, USA) was used for PCR. We designed specific primers for V5-His-ERRα (aa 193–423) as follows: F: 5′atggatccatgccagtgaatgcactggtgtc3′; R: 5′cgtctagagtccatcatggcctcgagcatc3′; Flag-p53 (aa 300–393) F: 5′ atggatcccccccagggagcactaagcga3′; R: 5′cctctagatgcatgctcgagtcag3′; and EGFP-luciferase-p53^72R^ F: 5′ atctcgaggatggaggagccgcagtcag3′; R: 5′ cgaccggtttagtctgagtcaggcccttctgtc3′. The V5-His-ERRα (aa 193–423), Flag-p53 (aa 300–393), and EGFP-luciferase-p53^72R^ PCR products were digested with BamHI-HF/Xba I and Xho I/Age I following instructions provided by the manufacturer (New England Biolabs, Ipswich MA, USA). The constructs were then inserted into the corresponding sites of pcDNA3.1/V5-His (Invitrogen by Thermo-Fisher Scientific), and pcDNA3/Flag and pLentipuro3/TO/V5/GW/EGFP/luciferase (Addgene Inc., Cambridge, MA, USA) to generate the encoding expression plasmids. Sanger DNA sequencing and the Blast program were used to confirm that the DNA was inserted into the corresponding sites.

### Lentiviral transductions

To generate stable knockdown ERRα cells, the lentiviral vector of ERRα (shERRα) or pLKO.1-puro shRNA Non-Target, control plasmid DNA (shMock), and packaging vectors (pMD2.0G and psPAX, Addgene Inc., Cambridge, MA, USA) were transduced into 293T cells by using iMFectin PolyDNA Transfection Reagent (GenDepot, Barker, TX, USA) following the manufacturer’s suggested protocols. To generate stable overexpression p53-mutant cells, the lentiviral vector of p53_^P72R^ containing EGFP and luciferase genes or pLentipuro3/TO/V5/GW/EGFP/luciferase, control plasmid DNA and packaging vectors (pMD2.0G and psPAX, Addgene Inc., Cambridge, MA, USA) were transduced into HCT-116^p53−/−^ and HCT-116^p53+/+^ cells, respectively, by using iMFectin PolyDNA Transfection Reagent (GenDepot, Barker, TX, USA) following the manufacturer’s suggested protocols. The transfection reagents were incubated with 293T, HCT-116^p53−/−^, and HCT-116^p53+/+^ cells in complete growth medium overnight, and then fresh medium with antibiotics (penicillin/streptomycin) was added. Viral supernatant fractions were collected at 48 h and filtered through a 0.45-μm syringe filter followed by infection into the appropriate cells together with 10 μg/mL polybrene (Sigma-Aldrich). After infection overnight, the medium was replaced with fresh complete growth medium containing the appropriate concentration ranging from 0.2 to 1 μg/mL puromycin (Gemini Bio Product) and cells incubated for an additional 1–3 weeks. The selected stable knockdown cells were used for further experiments.

### Quantification of mitochondrial DNA

Genomic DNA (gDNA) was extracted from HCT-116^p53+/+^ and HCT-116^p53−/−^ cells by using DNAzol® Reagent (Thermo Scientific) and quantified using the Human Mitochondrial DNA (mtDNA) Monitoring Primer Set (Takara Bio, Mountain View, CA, USA) according to manufacturer’s protocol. Primer sets for *ND1* and *ND5* were used for the detection of mtDNA, and the primer sets SLCO2B1 and *SERPINA1*were used for the detection of nuclear DNA. The quantitation of mtDNA copy number was performed using a 7500 FastDX (Applied Biosystems, MA, USA) and the power SYBR green PCR master mix (Applied Biosystem, Warrington WA1 4SR, UK).

### ATP production assay

Briefly, 10,000 cells were seeded in 96-well cell culture plates. HCT-116^p53+/+^ and HCT-116^p53−/−^ cells were treated either with DMSO as a negative control or with 15 μM XCT790 for 24 or 48 h. In the plate, the lysed cells were used for a luminescence ATP detection assay system by using ATPlite (PerkinElmer, Boston, MA, USA) with the Synergy™ Neo2 Multi-Mode Microplate Reader (BioTek, Winooski, VE, USA), following the manufacturer’s instructions. An ATP standard curve was generated, and the concentration of ATP was determined by the normalization of cell number.

### Immunofluorescence staining and apotome microscopy analysis

For mitochondrial staining, HCT-116^p53+/+^ and HCT-116^p53−/−^ live cells were first incubated with 0.3 μM MitoTracker Red CMXRos (Invitrogen, Eugene, Oregon, USA) for 30 min at 37 °C in 5% CO_2_. For co-localization and for mitochondrial staining, colon cancer cell lines were fixed with 3.7% formaldehyde, and then permeabilized in 300 μl of 0.5% Triton X-100. In general for immunostaining, after blocking the cover slips in 10% BSA for 1 h, the primary antibody was added overnight. The day after, Alexa Fluor 488-labeled or Alexa Fluor 568-labeled secondary antibodies (Thermo Scientific) were added (see Table S2). The DAPI-Fluoromount-G® solution (Electron Microscopy Sciences, Hatfield, PA, USA) was added for nuclei staining of the cells. Fluorescence-labeled proteins were analyzed using the Zeiss Fluorescent microscopy with apotome attachment and the Zeiss software and Axiocam cameras (Carl Zeiss Microscopy GmbH, Deutschland).

### Proliferation assay

Briefly, 20,000 cells were seeded in 24-well plates. The next day, cells were treated either with DMSO as a negative control or XCT790 (15 μM). Cells were incubated from 0 to 96 h. A survival assay was performed by using the cell proliferation Resazurin sodium salt reagent (Sigma-Aldrich), and the fluorescence excitation/emission wavelengths were measured at 545/595 nm with the Synergy™ Neo2 Multi-Mode Microplate Reader (BioTek, Winooski, VE, USA). Alternatively, 20,000 cells for non-targeting and knockdown of ERRα expression and for non-targeting and overexpression of p53^P72R^ were seeded in 24-well plates. Cells were incubated from 0 to 108 h, and a survival assay was performed by using the IncuCyteS3 live-cell imaging system (Essen BioScience, Tokyo, Japan), and then the IncuCyteS3:2019 software was used to quantify cell proliferation.

### Quantitative proteomics analysis by SWATH-MS

HCT-116^p53+/+^ and HCT-116^p53−/−^ cells were treated either with DMSO as a negative control or with 15 μM XCT790 for 48 h. Afterwards, cells were digested with trypsin and harvested in PBS. Cytosolic, membrane/organelle, and nuclear fractions were isolated using ProteoExtract® subcellular proteome Extraction kit (Millipore, Danvers, MA, USA) following the manufacturer’s suggested protocols. To screen differential protein expression across the samples in membrane/organelle fractions, MS/MS^ALL^ with SWATH^TM^ acquisition was conducted. Raw data was collected through information-dependent acquisition (IDA) and SWATH^TM^ acquisition Sciex TripleTOF™ 5600 with Eksigent 1D+ nano LC. The protein identification, MS peak extraction, and statistical analysis were performed with ProteinPilot^TM^ (version 4.5), PeakView^TM^ (version 2.2), and MarkerView^TM^ (version 1.2), respectively.

### Flow cytometry

For mitochondrial staining, HCT-116^p53+/+^ and HCT-116^p53−/−^ cells were incubated with 0.3 μM MitoTracker Red CMXRos (Invitrogen, Eugene, Oregon, USA) for 30 min at 37 °C in 5% CO_2_. Afterwards, cells were stained with 3 μM 4′,6-diamidino-2-phenylindole dihydrochloride (DAPI, Sigma-Aldrich) for 15 min at 37 °C in 5% CO_2_. For ROS measurement, HCT-116^p53+/+^ and HCT-116^p53−/−^ cells were incubated with 0.1 μM 2′,7′-dichlorofluorescin diacetate (H_2_DCFDA, Sigma-Aldrich) for 30 min at 37 °C in 5% CO_2_. Afterwards, cells were stained with 3 μM DAPI for 15 min at 37 °C in 5% CO_2_. All assays were performed using a BD FACSAria II flow cytometer (San Jose, CA, USA), and the BD FACSDiva Software version 8.0.2. Data were analyzed with the BD FlowJo version 10 software.

### Clonogenic assay

After XCT790 treatment, cell colony formation and cell survival were analyzed using colony-forming assay and staining with crystal violet and formaldehyde solution. Briefly, 40,000 cells were seeded in 6-well cell culture plates with 2 ml fresh growth medium. The next day, cells were treated either with DMSO as a negative control or XCT790 (15 μM). After treatment, cells were grown for another 7 days, and on day 7, cells were stained with crystal violet solution for 1 h and de-stained in plain water.

### Annexin V apoptotic cell staining and cell cycle assay

HCT-116^p53+/+^ and HCT-116^p53−/−^ cells were treated either with DMSO as a negative control or with 15 μM XCT790 for 48 h. Cells were then harvested and incubated with 1x Annexin V binding buffer containing Annexin V-FITC (BD Biosciences, Franklin Lake, NJ, USA) and propidium iodide (PI, Thermo Scientific) for 15 min in the dark at room temperature. Fractions of apoptotic cells were measured using flow cytometry. Also, for accessing the cell cycle progression of HCT-116^p53+/+^, HCT-116^p53−/−^, and DLD-1 cell lines, cells were harvested and incubated with propidium iodide (PI, Thermo Scientific) and RNase for 30 min in the dark at room temperature to measure cellular DNA content. All analyses were performed on LSRFortessa X-20 flow cytometer (San Jose, CA, USA) and the BD FACSDiva Software version 8.0.2. Data were analyzed with the BD FlowJo version 10 software.

### Quantitation and statistical analysis

Values are presented as mean values ± S.E.M. All experiments were conducted at least 2 to 4 times with representative data shown. Comparisons among values for all groups were determined by Student’s *t* test using the GraphPad Prism 8.0 software (San Diego, CA, USA). Differences between samples with a *p value of* ≤ 0.05 were considered statistically significant. For the analyses of proteomics, comparisons between groups were made using multiple *t* tests with a false discovery rate of 0.05.

## Supplementary Information

**Additional file 1: Figure S1**. Related to Fig. 1. A Purified ERRα proteins (100 μL) were loaded onto a Superose 6 size exclusion column. The elution profiles were recorded as absorbance at 280 nm. All the fractions from A7 to B10 were eluted from the column. B All fractions were loaded into an SDS–PAGE and then stained with Coomassie Brilliant Blue R-250 solution. C Western blot analysis was performed using anti-ERRα and anti-p53. D 293T cell line *(left)* and DLD-1 *(right)* colon cancer cell line were used to perform endogenous IP followed by IB analysis with anti-ERRα, anti-PGC-1α, and anti-GAPDH.

**Additional file 2: Figure S2**. Related to Fig. 1h. A The p53 CTD (Lys370 – Asp391) in green is superposed on the box3-peptide of PGC1α (Gln203 – Asp224) shown in red. The ERRα LBD is shown in yellow. B The box3-peptide of PGC1α is removed from the complex shown in (A). C Zoomed in the interface between the p53 CTD and the box3-peptide of PGC1α showing three potential non-bonding interactions. D Electrostatic surface representation of ERRα at the binding interface with p53, where the red color indicating negatively charged region, blue color being the positively charged region, and the in-between gray color being the hydrophobic region. Here, the hydrophobic residue Leu383 from p53 is trapped within a hydrophobic pocket formed by several hydrophobic residues of ERRα at the binding interface. E Sequence alignment between the ERα LBD (Met192-Tyr389) and the ERRα LBD (Val225-Tyr414). F Superposition of the ERα LBD (in orange) and the ERRα LBD (in green). G Sequence alignment between the box3-peptide of PGC1α (Gln203 – Asp224) and the p53 CTD (Lys370 – Asp391). H Non-bonding interactions between p53 and ERRα at the interface.

**Additional file 3: Figure S3**. Related to Fig. 2. A IB analysis was conducted using anti-ERRα, anti-p53, and anti-actin in HCT-116^p53+/+^ and HCT-116^p53-/-^ cells. B IF analysis to detect COX-4 and VDAC1 was conducted in HCT-116^p53+/+^ and HCT-116^p53-/-^ cells. Enlarged panels represent selected digitally enlarged portions of parent images to enhance the visibility of COX-4 and VDAC1. Co-localization of COX-4 and VDAC1 was quantified (as % overlay); scale bar, 50 μm. C HCT-116^p53-/-^cell growth was analyzed. D Cell cycle was assessed by PI staining and flow cytometry in DLD-1 cells as described in Methods. E IB analysis was conducted with anti-ERRα, anti-p27^(KIP1)^, anti-p21^(WAF1/CIP1)^, anti-HSP-70, anti-p15^(INK4B)^, anti-cyclin D1, anti-cyclin E, and anti-actin in DLD-1 cells. All cells were stably transduced with lentiviral constructs expressing an shRNA specific to ERRα (shERRα#) or an shRNA non-targeting construct (shMock). The data are shown as means ± S.D. (n = 2-4). The *p* value was calculated using a two-tailed Student’s t test. * *p* ≤ 0.05; ** *p* ≤ 0.01; *** *p* ≤ 0.001; **** *p* ≤ 0.0001; n.s., not significant.

**Additional file 4: Figure S4**. Related to Fig. 3 A HCT-116^p53+/+^ cells were treated for 48 h with XCT790 (15 μM) or vehicle (DMSO) and transiently transfected with pCMV flag ERRα or pcDNA3 empty vector (mock). IB analysis was conducted with anti-ERRα, anti-p53, and anti-actin. B-C Enriched KEGG pathways up-regulated and down-regulated obtained by STRING analysis of the membrane/organelle purified proteins fraction comparing (ii) absence of ERRα with (i) presence of ERRα and p53 or comparing (iii) absence of p53 with (i) presence of ERRα and p53. Comparisons between groups were made using multiple t tests with a False Discovery Rate of *p* ≤ 0.05.

**Additional file 5: Figure S5**. Related to Fig. 5. A Cell cycle progression was assessed by PI staining and flow cytometry as described in Methods. B IB analysis was performed with anti-ERRα, anti-p53, anti-p21^(WAF1/CIP1)^, anti-cyclin D1, and anti-actin. C Cell growth was analyzed. All experiments were conducted using HCT-116^p53+/+^ and HCT-116^p53-/-^ cells treated with XCT790 (15 μM) or vehicle (DMSO). The data are shown as means ± S.D. (n = 2-4). The *p* value was calculated using a two-tailed Student’s t test. * *p* ≤ 0.05; ** *p* ≤ 0.01; *** *p* ≤ 0.001; **** *p* ≤ 0.0001; n.s., not significant.

**Additional file 6: Figure S6**. Related to Fig. 6. A Overall p53 mutational spectrum was performed for 37 colon cancer patients. B IB analysis was conducted using anti-p53 and anti-GAPDH in HCT-116^p53+/+^ and HCT-116^p53-/-^ cells. Images show the GFP signal. C A random toxicity study was performed. All animals were euthanized and liver and spleen were extracted and weighed (n = 4). D At the end of the treatment period, all animals were euthanized and liver and spleen were extracted and weighed (n = 7).

**Additional file 7: Table S1**. List of proteins identified through mass spectrometry-based proteomics. All experiments were conducted using HCT-116^p53+/+^ and HCT-116^p53-/-^ cells treated with XCT790 (15 μM) or vehicle (DMSO).

**Additional file 8: Table S2**. List of antibodies, chemicals, commercial assays, cell lines, oligonucleotides, recombinant DNA, software, and algorithms.

## Data Availability

Further information and requests for resources and or reagents can be directed to and will be fulfilled by the lead contact, Zigang Dong: dongzg@zzu.edu.cn

## Abbreviations

ERRα: Estrogen-related receptor alpha
LBD: Ligand-binding domain
CTD: C-terminal domain
ROS: Reactive oxygen species
MMP: Mitochondrial membrane permeabilization
mtOxPhos: Oxidative phosphorylation
NRs: Nuclear receptors
ERRβ and ERRγ: Estrogen-related receptor beta and gamma
ERα: Estrogen receptor alpha
PGC-1α: Peroxisome proliferator-activated receptor gamma coactivator 1-alpha
TFs: Transcription factors
GOF: Gain-of-functions
SNP: Single base-pair substitutions
YAP: Yes-associated protein
NRF2: Nuclear factor erythroid 2–related factor 2
COX-4: Cytochrome *c* oxidase subunit IV
VDAC1: Voltage-dependent anion channel 1
KEGG: Kyoto Encyclopedia of Genes and Genomes
AIF: Apoptosis-inducing factor
PARP: Poly (ADP-ribose) polymerase
