# Supplementary figures and images for: Estrogen-related receptor alpha directly binds to p53 and cooperatively controls colon cancer growth through the regulation of mitochondrial biogenesis and function
Source: Cancer Metab. 2020 Dec 10;8:28. doi: 10.1186/s40170-020-00234-5 (PMC7731476; doi:10.1186/s40170-020-00234-5)

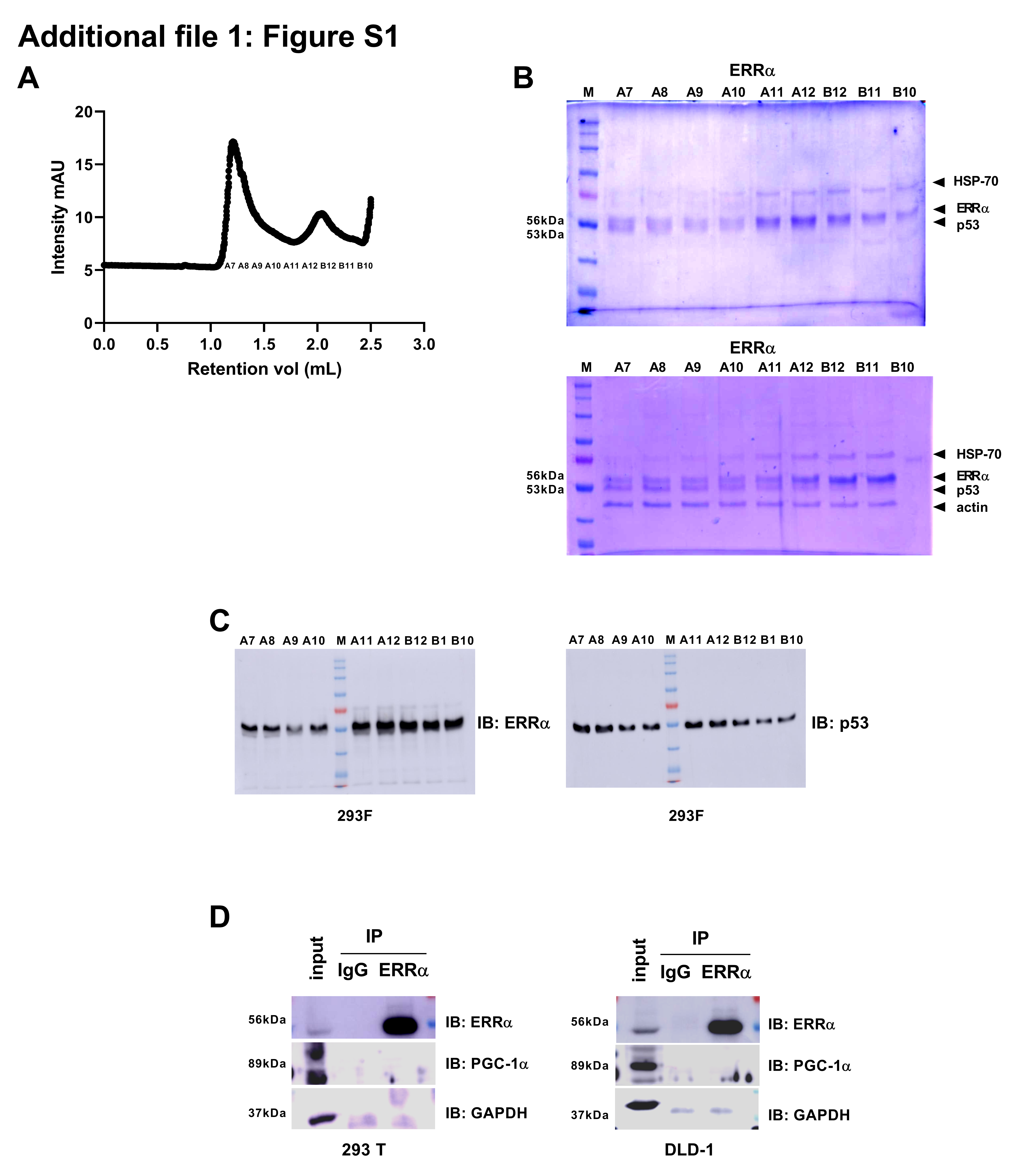

Supplement: Supplementary file 1 — Additional file 1: Figure S1. Related to Fig. 1. A Purified ERRα proteins (100 μL) were loaded onto a Superose 6 size exclusion column. The elution profiles were recorded as absorbance at 280 nm. All the fractions from A7 to B10 were eluted from the column. B All fractions were loaded into an SDS–PAGE and then stained with Coomassie Brilliant Blue R-250 solution. C Western blot analysis was performed using anti-ERRα and anti-p53. D 293T cell line (left) and DLD-1 (right) colon cancer cell line were used to perform endogenous IP followed by IB analysis with anti-ERRα, anti-PGC-1α, and anti-GAPDH. [file 40170_2020_234_MOESM1_ESM.tif]

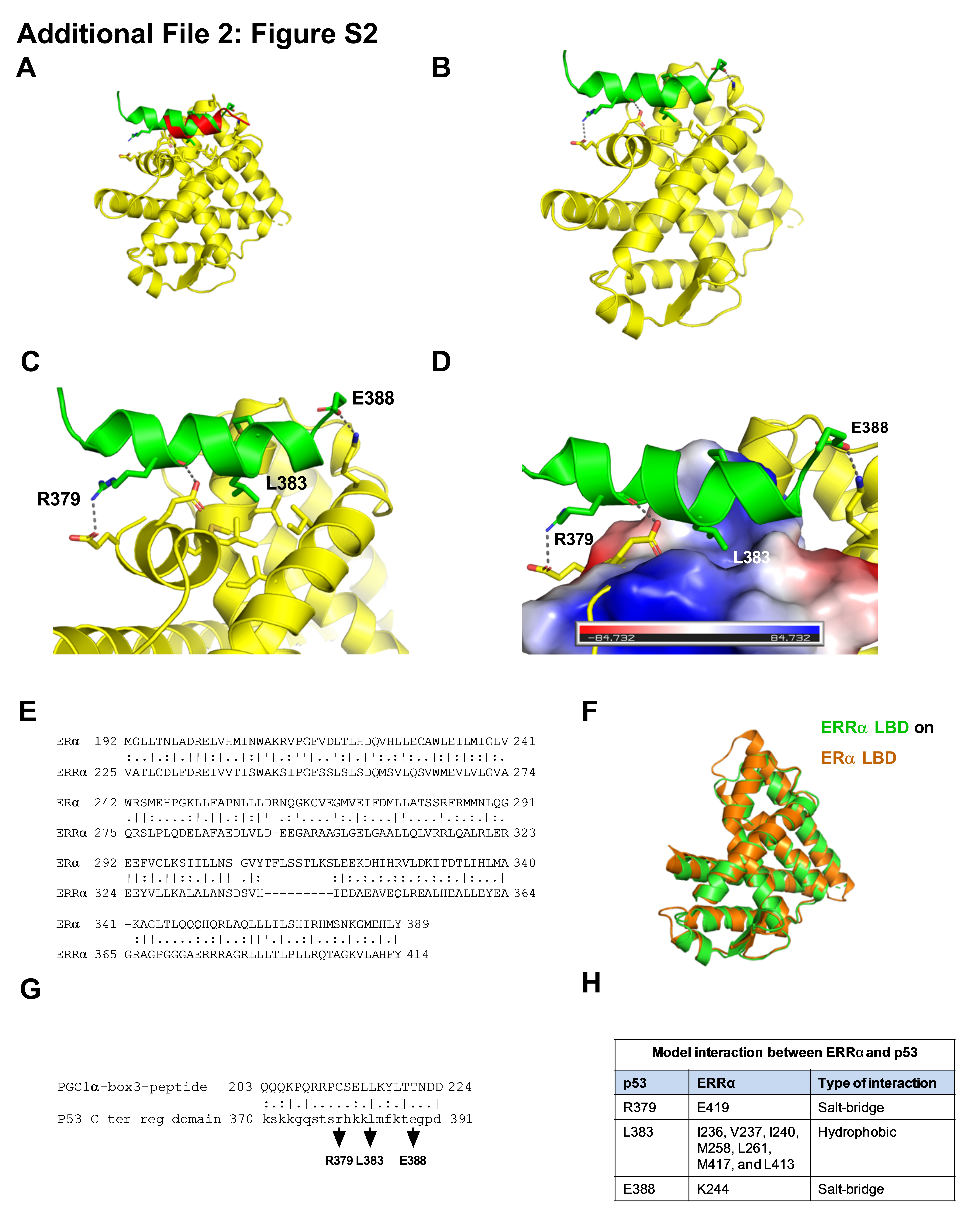

Supplement: Supplementary file 2 — Additional file 2: Figure S2. Related to Fig. 1h. A The p53 CTD (Lys370 – Asp391) in green is superposed on the box3-peptide of PGC1α (Gln203 – Asp224) shown in red. The ERRα LBD is shown in yellow. B The box3-peptide of PGC1α is removed from the complex shown in (A). C Zoomed in the interface between the p53 CTD and the box3-peptide of PGC1α showing three potential non-bonding interactions. D Electrostatic surface representation of ERRα at the binding interface with p53, where the red color indicating negatively charged region, blue color being the positively charged region, and the in-between gray color being the hydrophobic region. Here, the hydrophobic residue Leu383 from p53 is trapped within a hydrophobic pocket formed by several hydrophobic residues of ERRα at the binding interface. E Sequence alignment between the ERα LBD (Met192-Tyr389) and the ERRα LBD (Val225-Tyr414). F Superposition of the ERα LBD (in orange) and the ERRα LBD (in green). G Sequence alignment between the box3-peptide of PGC1α (Gln203 – Asp224) and the p53 CTD (Lys370 – Asp391). H Non-bonding interactions between p53 and ERRα at the interface. [file 40170_2020_234_MOESM2_ESM.tif]

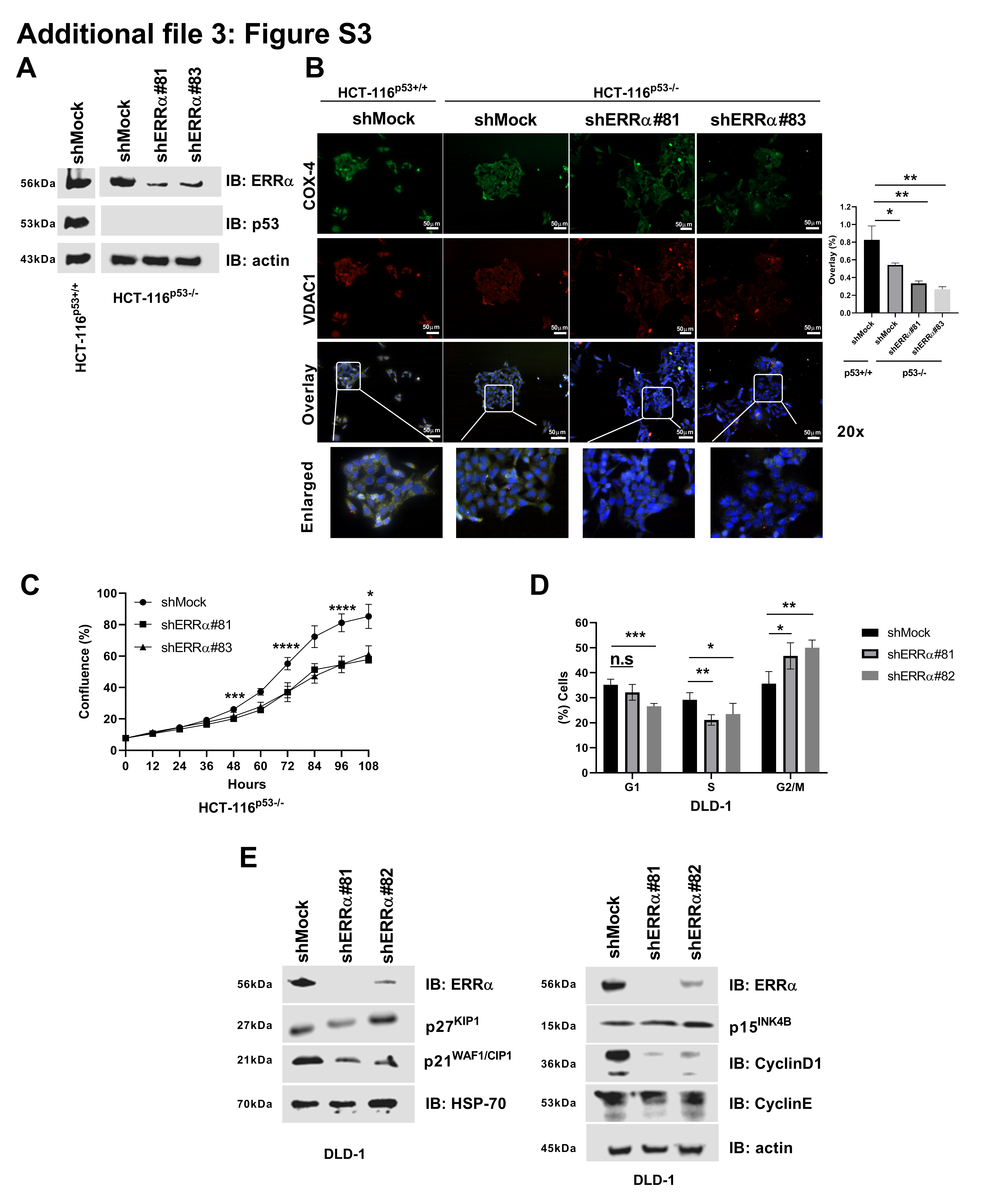

Supplement: Supplementary file 3 — Additional file 3: Figure S3. Related to Fig. 2. A IB analysis was conducted using anti-ERRα, anti-p53, and anti-actin in HCT-116p53+/+ and HCT-116p53-/- cells. B IF analysis to detect COX-4 and VDAC1 was conducted in HCT-116p53+/+ and HCT-116p53-/- cells. Enlarged panels represent selected digitally enlarged portions of parent images to enhance the visibility of COX-4 and VDAC1. Co-localization of COX-4 and VDAC1 was quantified (as % overlay); scale bar, 50 μm. C HCT-116p53-/-cell growth was analyzed. D Cell cycle was assessed by PI staining and flow cytometry in DLD-1 cells as described in Methods. E IB analysis was conducted with anti-ERRα, anti-p27(KIP1), anti-p21(WAF1/CIP1), anti-HSP-70, anti-p15(INK4B), anti-cyclin D1, anti-cyclin E, and anti-actin in DLD-1 cells. All cells were stably transduced with lentiviral constructs expressing an shRNA specific to ERRα (shERRα#) or an shRNA non-targeting construct (shMock). The data are shown as means ± S.D. (n = 2-4). The p value was calculated using a two-tailed Student’s t test. * p ≤ 0.05; ** p ≤ 0.01; *** p ≤ 0.001; **** p ≤ 0.0001; n.s., not significant. [file 40170_2020_234_MOESM3_ESM.tif]

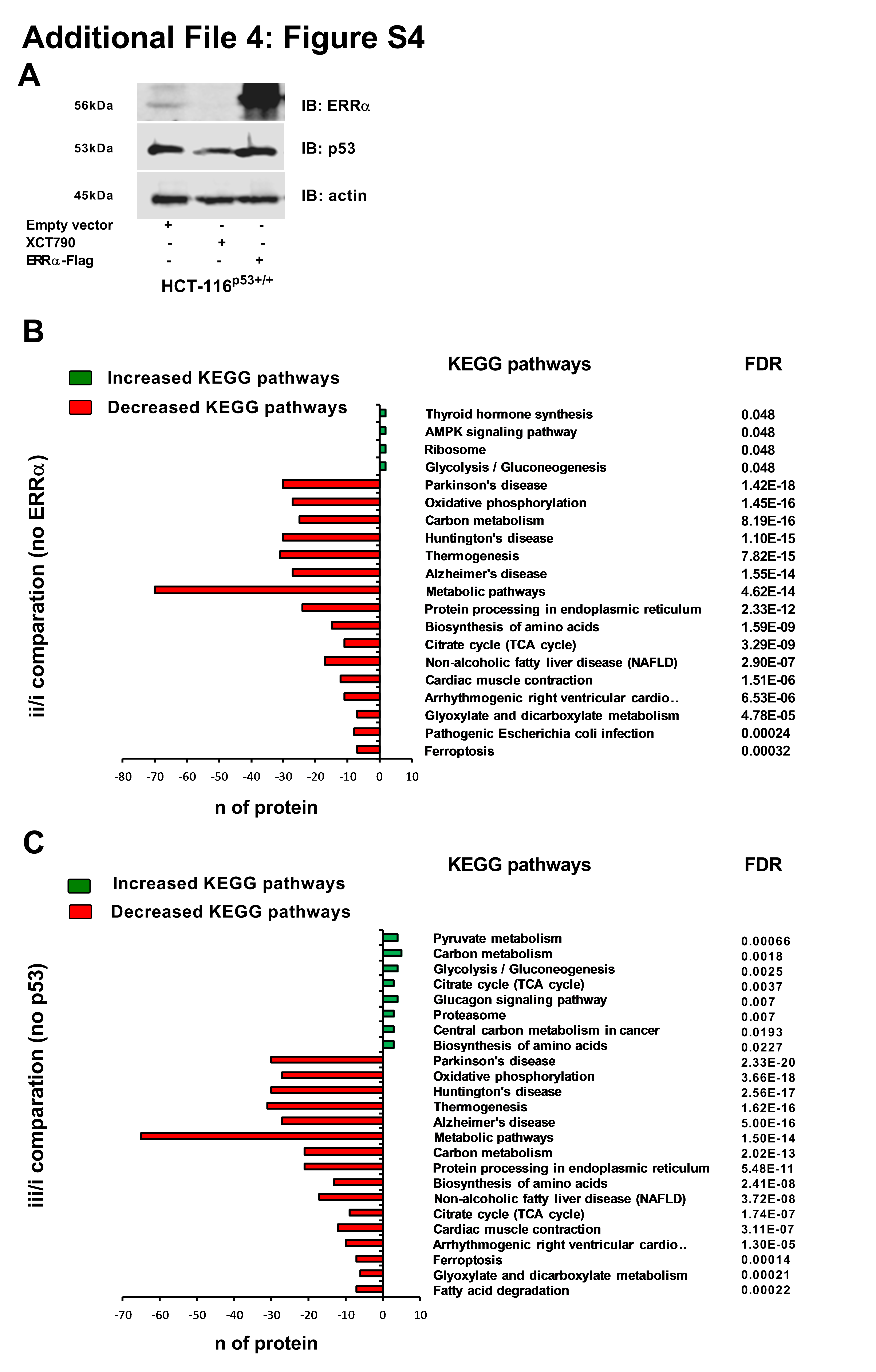

Supplement: Supplementary file 4 — Additional file 4: Figure S4. Related to Fig. 3 A HCT-116p53+/+ cells were treated for 48 h with XCT790 (15 μM) or vehicle (DMSO) and transiently transfected with pCMV flag ERRα or pcDNA3 empty vector (mock). IB analysis was conducted with anti-ERRα, anti-p53, and anti-actin. B-C Enriched KEGG pathways up-regulated and down-regulated obtained by STRING analysis of the membrane/organelle purified proteins fraction comparing (ii) absence of ERRα with (i) presence of ERRα and p53 or comparing (iii) absence of p53 with (i) presence of ERRα and p53. Comparisons between groups were made using multiple t tests with a False Discovery Rate of p ≤ 0.05. [file 40170_2020_234_MOESM4_ESM.tif]

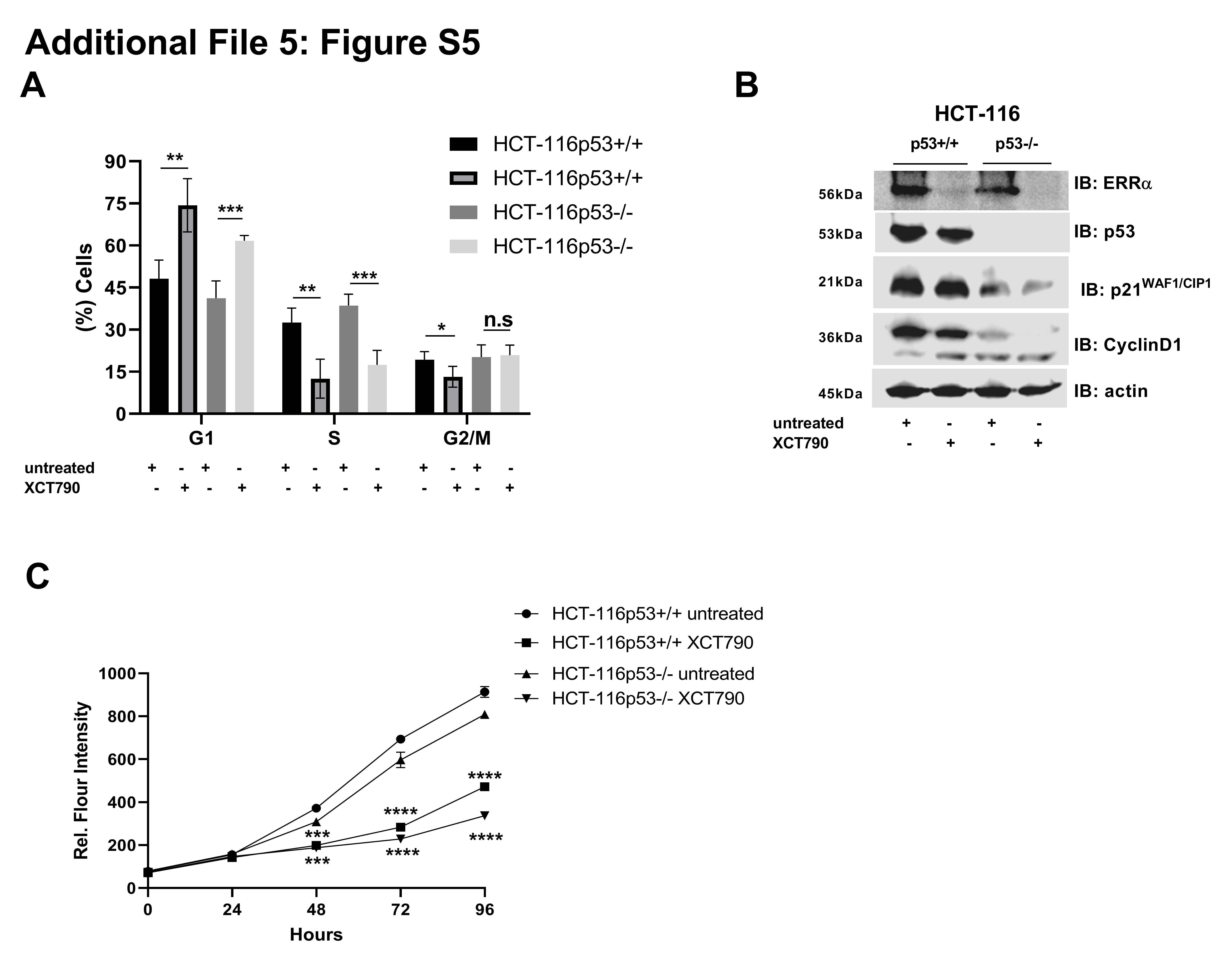

Supplement: Supplementary file 5 — Additional file 5: Figure S5. Related to Fig. 5. A Cell cycle progression was assessed by PI staining and flow cytometry as described in Methods. B IB analysis was performed with anti-ERRα, anti-p53, anti-p21(WAF1/CIP1), anti-cyclin D1, and anti-actin. C Cell growth was analyzed. All experiments were conducted using HCT-116p53+/+ and HCT-116p53-/- cells treated with XCT790 (15 μM) or vehicle (DMSO). The data are shown as means ± S.D. (n = 2-4). The p value was calculated using a two-tailed Student’s t test. * p ≤ 0.05; ** p ≤ 0.01; *** p ≤ 0.001; **** p ≤ 0.0001; n.s., not significant. [file 40170_2020_234_MOESM5_ESM.tif]

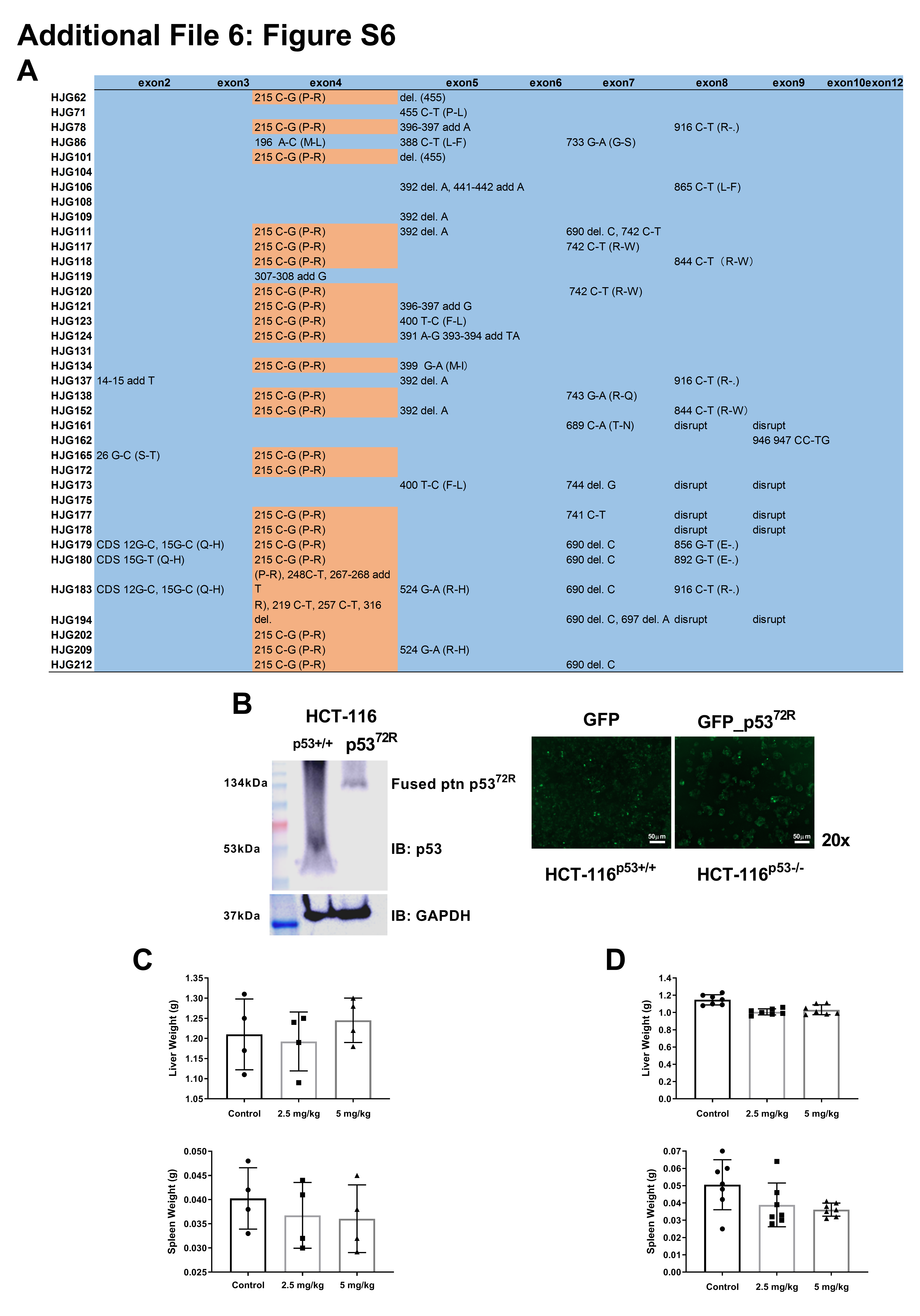

Supplement: Supplementary file 6 — Additional file 6: Figure S6. Related to Fig. 6. A Overall p53 mutational spectrum was performed for 37 colon cancer patients. B IB analysis was conducted using anti-p53 and anti-GAPDH in HCT-116p53+/+ and HCT-116p53-/- cells. Images show the GFP signal. C A random toxicity study was performed. All animals were euthanized and liver and spleen were extracted and weighed (n = 4). D At the end of the treatment period, all animals were euthanized and liver and spleen were extracted and weighed (n = 7). [file 40170_2020_234_MOESM6_ESM.tif]
