# Supplementary material for: Estrogen-related receptor alpha directly binds to p53 and cooperatively controls colon cancer growth through the regulation of mitochondrial biogenesis and function
Source: Cancer Metab. 2020 Dec 10;8:28. doi: 10.1186/s40170-020-00234-5 (PMC7731476; doi:10.1186/s40170-020-00234-5)
Supplement: Supplementary file 8 — Additional file 8: Table S2. List of antibodies, chemicals, commercial assays, cell lines, oligonucleotides, recombinant DNA, software, and algorithms. [file 40170_2020_234_MOESM8_ESM.docx]

**Table S2.** List of antibodies, chemicals, commercial assays, cell lines, oligonucleotides, recombinant DNA, software, and algorithms.

| REAGENT or RESOURCE | SOURCE | IDENTIFIER |
| --- | --- | --- |
| **Antibodies** | | |
| Rabbit monoclonal anti-ERRα | Cell Signaling Technology | Cat#13826 |
| Rabbit polyclonal anti-p53 | Cell Signaling Technology | Cat#9282 |
| Rabbit monoclonal anti-AIF | Cell Signaling Technology | Cat#5318 |
| Mouse monoclonal anti-Smac/Diablo | Cell Signaling Technology | Cat#2954 |
| Rabbit polyclonal anti-Cleaved caspase-3 | Cell Signaling Technology | Cat#9661 |
| Rabbit monoclonal anti-Cleaved PARP | Cell Signaling Technology | Cat#5625 |
| Rabbit monoclonal anti-p21^WAF1/CIP1^ | Cell Signaling Technology | Cat#2947S |
| Rabbit polyclonal anti-p15^INK4^ | Cell Signaling Technology | Cat#4822_ |
| Mouse monoclonal anti-ERRα | Santa Cruz Biotechnology | Cat#sc-65718 |
| Mouse monoclonal anti-ERRγ | Santa Cruz Biotechnology | Cat#sc-393969 |
| Rabbit polyclonal anti-ERα | Santa Cruz Biotechnology | Cat#sc-543 |
| Mouse monoclonal anti-p53 | Santa Cruz Biotechnology | Cat#sc-126 |
| Mouse monoclonal anti-GAPDH | Santa Cruz Biotechnology | Cat#sc-32233 |
| Mouse monoclonal anti-β-actin | Santa Cruz Biotechnology | Cat#sc-47778 |
| Goat polyclonal anti-VDAC1 | Santa Cruz Biotechnology | Cat#sc-8828 |
| Mouse monoclonal anti-Lamin B | Santa Cruz Biotechnology | Cat#sc-374015 |
| Mouse monoclonal anti-α-tubulin | Santa Cruz Biotechnology | Cat#sc-69969 |
| Mouse monoclonal anti-HSP-70 | Santa Cruz Biotechnology | Cat#sc-24 |
| Mouse monoclonal anti-cytochrome c | Santa Cruz Biotechnology | Cat#sc-13156 |
| Mouse monoclonal anti-Caspase-3 | Santa Cruz Biotechnology | Cat#sc-56053 |
| Mouse monoclonal anti-Cyclin D1 | Santa Cruz Biotechnology | Cat#sc-8396 |
| Mouse monoclonal anti-Cyclin E | Santa Cruz Biotechnology | Cat#sc-248 |
| Mouse monoclonal anti-p27^KIP^ | Santa Cruz Biotechnology | Cat#sc-393380 _ |
| Normal mouse IgG | Santa Cruz Biotechnology | Cat#sc-2025 |
| Rabbit polyclonal anti-PGC-1 | EMD-Millipore-Sigma | Cat#AB3242 |
| Mouse monoclonal anti-HSP-90 | Enzo Life Science | Cat#ADI-SPA-830-D |
| **Antibodies** |  |  |
| Total OXPHOS WB cocktail | Abcam | Cat#Ab110413 |
| Rabbit polyclonal anti-COX-4 | StressMarq Biosciences | Cat#SPC-688S |
| Goat anti-Rabbit IgG (H+L) Secondary antibody, Alexa Fluor Plus 488 | Thermo Scientific | Cat#A32731 |
| Goat anti-Mouse IgG (H+L) Secondary antibody, Alexa Fluor Plus 488 | Thermo Scientific | Cat# A32723 |
| Donkey anti-Goat Rabbit IgG (H+L) Secondary antibody, Alexa Fluor Plus 568 | Thermo Scientific | Cat#A-11057 |
| Mouse monoclonal Anti-Flag® M2 | Sigma-Aldrich | Cat#F3165-1MG |
| **Chemicals, Peptides, and Recombinant Proteins** | | |
| IMfectin Poly DNA Transfection Reagent | GenDEPOT | Cat#I7200 |
| Protein G PLUS-Agarose | Santa Cruz Biotechnology | Cat#sc-2002 |
| XCT790 | APExBIO | Cat#B3238 |
| Resazurin sodium salt | Sigma-Aldrich | Cat#R7017 |
| 2′,7′-Dichlorofluorescin diacetate (H_2_DCFDA) | Sigma-Aldrich | Cat#D6883 |
| DAPI | Sigma-Aldrich | Cat#D9542 |
| N-Acetyl-Cysteine | Sigma-Aldrich | Cat#A7250 |
| Crystal Violet | Sigma-Aldrich | Cat#C6158 |
| Polyethylenimine (PEI), linear | Sigma-Aldrich | Cat#764582-1G |
| ANTI-FLAG^®^M2 Affinity Gel | Millipore | Cat#A2220 |
| 3X FLAG^®^Peptide | Millipore | Cat#F4799 |
| MitoTracker Red CMXRos | Thermo Scientific | Cat#M7512 |
| Annexin V-FITC | BD Biosciences | Cat# 556420 |
| Propidium iodide | Thermo Scientific | Cat#P3566 |
| Hydrogen Peroxide 30% | Thermo Scientific | Cat#H325-100 |
| DAPI-Fluoromount-G^®^ | Electron Microscopy Sciences | Cat#17984-24 |
| Polybrene | Sigma-Aldrich | Cat#TR-1003-G |
| **Chemicals, Peptides, and Recombinant Proteins** |  |  |
| Puromycin | Gemini Bio Product | Cat#400-128P |
| Insulin – human recombinant | GenDEPOT | Cat#I4500-025 |
| Hydrocortisone | GenDEPOT | Cat#CA051-001 |
| Coomassie Brilliant Blue R-250 | Thermo Scientific | Cat#20278 |
| **Critical Commercial Assays** | | |
| ATPlite | PerkinElmer | Cat#6016943 |
| ProteoExtract subcellular proteome Extraction kit | Millipore Sigma | Cat#539790 |
| Duolink in situ starter kit | Sigma-Aldrich | Cat#DUO92101-1KT |
| DNAzol® Reagent | Thermo Scientific | Cat#10503-027 |
| PrimeSTAR® GXL DNA Polymerase | Takara Bio | Cat#R050B |
| Human Mitochondrial DNA (mtDNA) Monitoring Primer Set | Takara Bio | Cat#7246 |
| DC^TM^ protein assay | Bio-Rad | Cat#5000111 |
| SYBR green PCR master mix | Applied biosystem | Cat#4367659 |
| **Experimental Models: Cell Lines** | | |
| Human: Free-Style^TM^ 293-F cells | Thermo Scientific | Cat#R79007 |
| Human:293T | ATCC | Cat#CRL-3216 |
| Human: HCT-116^p53+/+^ | ATCC | Cat#CCL-247 |
| Human:HCT-116^p53-/-^ | [1] | N/A |
| Human:HCT-15 | ATCC | Cat#CCL-225 |
| Human:DLD-1 | ATCC | Cat#CCL-221 |
| Human: CCD-18Co | ATCC | Cat#CRL-1459 |
| Human:Lim1215 | ECACC | Cat#10092301 |
| Human:HT-29 | ATCC | Cat#HTB-38 |
| Human:SW480 | ATCC | Cat#CCL-228 |
| Human:WiDr | ATCC | Cat#CCL-218 |
| Human:Caco-2 | ATCC | Cat# HTB-37 |
| **Oligonucleotides** | | |
| ERRα Forward:  atggatccatgccagtgaatgcactggtgtc | IDT primer | N/A |
| ERRα Reverse: cgtctagagtccatcatggcctcgagcatc | IDT primer | N/A |
| p53 Forward:  atggatcccccccagggagcactaagcga | IDT primer | N/A |
| p53 Reverse:  cctctagatgcatgctcgagtcag | IDT primer | N/A |
| EGFP-luciferase- p53^72R^ Forward  Atctcgaggatggaggagccgcagtcag | IDT primer | N/A |
| EGFP-luciferase- p53^72R^ Reverse  cgaccggtttagtctgagtcaggcccttctgtc | IDT primer | N/A |
| **Recombinant DNA** | | |
| Plasmid: pCMV flag ERRα | Addgene | Cat#10975 |
| Plasmid: pcDNA3 flag p53 | Addgene | Cat#10838 |
| Plasmid: pcDNA3.1 V5-His | Invitrogen | Cat#V81020 |
| Plasmid: pLentipuro3/TO/V5/GW/EGFP/luciferase | Addgene | Cat#119816 |
| Plasmid: pMD2.0G | Addgene | Cat#12259 |
| Plasmid: psPAX | Addgene | Cat#12260 |
| Plasmid: pLKO.1 shRNA non-targeting | UMGC RNAi Core Database | N/A |
| Plasmid: pLKO.1 shRNA targeting human ERRα #79 | UMGC RNAi Core Database | N/A |
| Plasmid: pLKO.1 shRNA targeting human ERRα #81 | UMGC RNAi Core Database | N/A |
| Plasmid: pLKO.1 shRNA targeting human ERRα #82 | UMGC RNAi Core Database | N/A |
| Plasmid: pLKO.1 shRNA targeting human ERRα #83 | UMGC RNAi Core Database | N/A |
| **Software and Algorithms** | | |
| ImageJ | Fiji | https://imagej.nih.gov/ij/ |
| GraphPad Prism 8 | GraphPad Software | https://www.graphpad.com/scientificsoftware/  prism/ |

Reference

1. Sur S, Pagliarini R, Bunz F, Rago C, Diaz LA, Kinzler KW, Vogelstein B, Papadopoulos N: **A panel of isogenic human cancer cells suggests a therapeutic approach for cancers with inactivated p53.** *Proc Natl Acad Sci U S A* 2009, **106:**3964-3969.
